# Exposure of progressive immune dysfunction by SARS-CoV-2 mRNA vaccination in patients with chronic lymphocytic leukemia: A prospective cohort study

**DOI:** 10.1371/journal.pmed.1004157

**Published:** 2023-06-29

**Authors:** Kai Qin, Kazuhito Honjo, Scott Sherrill-Mix, Weimin Liu, Regina M. Stoltz, Allisa K. Oman, Lucinda A. Hall, Ran Li, Sarah Sterrett, Ellen R. Frederick, Jeffrey R. Lancaster, Mayur Narkhede, Amitkumar Mehta, Foluso J. Ogunsile, Rima B. Patel, Thomas J. Ketas, Victor M. Cruz Portillo, Albert Cupo, Benjamin M. Larimer, Anju Bansal, Paul A. Goepfert, Beatrice H. Hahn, Randall S. Davis

**Affiliations:** 1 Department of Medicine, University of Alabama at Birmingham, Birmingham, Alabama, United States of America; 2 Department of Medicine, University of Pennsylvania, Philadelphia, Pennsylvania, United States of America; 3 Department of Radiology, University of Alabama at Birmingham, Birmingham, Alabama, United States of America; 4 O’Neal Comprehensive Cancer Center, University of Alabama at Birmingham, Birmingham, Alabama, United States of America; 5 Department of Microbiology and Immunology, Weill Medical College of Cornell University, New York, New York, United States of America; 6 Department of Microbiology, University of Alabama at Birmingham, Birmingham, Alabama, United States of America; 7 Department of Microbiology, University of Pennsylvania, Philadelphia, Pennsylvania, United States of America; 8 Department of Biochemistry and Molecular Genetics, University of Alabama at Birmingham, Birmingham, Alabama, United States of America; Peter MacCallum Cancer Centre, AUSTRALIA

## Abstract

**Background:**

Patients with chronic lymphocytic leukemia (CLL) have reduced seroconversion rates and lower binding antibody (Ab) and neutralizing antibody (NAb) titers than healthy individuals following Severe Acute Respiratory Syndrome Coronavirus 2 (SARS-CoV-2) mRNA vaccination. Here, we dissected vaccine-mediated humoral and cellular responses to understand the mechanisms underlying CLL-induced immune dysfunction.

**Methods and findings:**

We performed a prospective observational study in SARS-CoV-2 infection-naïve CLL patients (*n* = 95) and healthy controls (*n* = 30) who were vaccinated between December 2020 and June 2021. Sixty-one CLL patients and 27 healthy controls received 2 doses of the Pfizer-BioNTech BNT162b2 vaccine, while 34 CLL patients and 3 healthy controls received 2 doses of the Moderna mRNA-1273 vaccine. The median time to analysis was 38 days (IQR, 27 to 83) for CLL patients and 36 days (IQR, 28 to 57) for healthy controls. Testing plasma samples for SARS-CoV-2 anti-spike and receptor-binding domain Abs by enzyme-linked immunosorbent assay (ELISA), we found that all healthy controls seroconverted to both antigens, while CLL patients had lower response rates (68% and 54%) as well as lower median titers (23-fold and 30-fold; both *p* < 0.001). Similarly, NAb responses against the then prevalent D614G and Delta SARS-CoV-2 variants were detected in 97% and 93% of controls, respectively, but in only 42% and 38% of CLL patients, who also exhibited >23-fold and >17-fold lower median NAb titers (both *p* < 0.001). Interestingly, 26% of CLL patients failed to develop NAbs but had high-titer binding Abs that preferentially reacted with the S2 subunit of the SARS-CoV-2 spike. Since these patients were also seropositive for endemic human coronaviruses (HCoVs), these responses likely reflect cross-reactive HCoV Abs rather than vaccine-induced de novo responses. CLL disease status, advanced Rai stage (III-IV), elevated serum beta-2 microglobulin levels (β2m >2.4 mg/L), prior therapy, anti-CD20 immunotherapy (<12 months), and intravenous immunoglobulin (IVIg) prophylaxis were all predictive of an inability to mount SARS-CoV-2 NAbs (all *p* ≤ 0.03). T cell response rates determined for a subset of participants were 2.8-fold lower for CLL patients compared to healthy controls (0.05, 95% CI 0.01 to 0.27, *p* < 0.001), with reduced intracellular IFNγ staining (*p* = 0.03) and effector polyfunctionality (*p* < 0.001) observed in CD4^+^ but not in CD8^+^ T cells. Surprisingly, in treatment-naïve CLL patients, BNT162b2 vaccination was identified as an independent negative risk factor for NAb generation (5.8, 95% CI 1.6 to 27, *p* = 0.006). CLL patients who received mRNA-1273 had 12-fold higher (*p* < 0.001) NAb titers and 1.7-fold higher (6.5, 95% CI 1.3 to 32, *p* = 0.02) response rates than BNT162b2 vaccinees despite similar disease characteristics. The absence of detectable NAbs in CLL patients was associated with reduced naïve CD4^+^ T cells (*p* = 0.03) and increased CD8^+^ effector memory T cells (*p* = 0.006). Limitations of the study were that not all participants were subjected to the same immune analyses and that pre-vaccination samples were not available.

**Conclusions:**

CLL pathogenesis is characterized by a progressive loss of adaptive immune functions, including in most treatment-naïve patients, with preexisting memory being preserved longer than the capacity to mount responses to new antigens. In addition, higher NAb titers and response rates identify mRNA-1273 as a superior vaccine for CLL patients.

## Abstract 2

In a prospective cohort study, Kai Qin and colleagues investigate underlying mechanisms of immune dysfunction in CLL, by measuring humoral and cellular immune responses to SARS-CoV-2 mRNA vaccination in patients with varying degrees of disease progression.

## Author summary

**Why was this study done?:**

- Chronic lymphocytic leukemia (CLL) patients are immunocompromised, have a high risk of acquiring life-threatening infections, and generally respond poorly to vaccination.
- CLL patients also have low seroconversion rates and binding antibody (Ab) and neutralizing antibody (NAb) titers following Severe Acute Respiratory Syndrome Coronavirus 2 (SARS-CoV-2) mRNA vaccination.
- To probe the underlying mechanisms of immune dysfunction in CLL, we studied both humoral and cellular immune responses to SARS-CoV-2 mRNA vaccination in patients with varying degrees of disease progression.

**What did the researchers do and find?:**

- We enrolled SARS-CoV-2 infection-naive healthy participants (*n* = 30) and CLL patients (*n* = 95) who received either 2 doses of the Pfizer-BioNTech BNT162b2 or the Moderna mRNA-1273 vaccine.
- We found spike-binding Abs and NAbs in 100% and 97% of healthy controls, respectively, but in only 68% and 42% of CLL patients, who also had much lower titers. T cell responses were found in 91% of healthy controls compared to 33% of CLL patients.
- Approximately 26% of CLL patients who lacked SARS-CoV-2 NAbs preferentially reacted with the S2 subunit of the SARS-CoV-2 spike, suggesting cross-reactivity with preexisting common coronavirus Abs.
- mRNA-1273 induced higher NAb response rates and titers than BNT162b2 in treatment-naïve CLL vaccinees despite similar disease progression.

**What do these findings mean?:**

- Dissection of SARS-CoV-2 vaccine-induced humoral and cellular responses revealed a progressive deterioration of adaptive immune functions, even in CLL patients who had never received therapy.
- The “seroconversion” of over a quarter of CLL vaccinees likely reflects the presence of non-protective common coronavirus Abs that cross-react with the SARS-CoV-2 spike protein.
- Higher Ab response rates and NAb titers indicate that mRNA-1273 may be more beneficial for CLL patients.
- CLL patients and other immunocompromised populations may benefit from alternative vaccine regimens, which should be evaluated in clinical trials.

## Introduction

Chronic lymphocytic leukemia (CLL) is the most prevalent leukemia in western countries and mainly affects the elderly, with a median age at diagnosis of 70 years [1]. Because the natural progression of this B cell malignancy as well as its treatments weakens innate and adaptive immunity, infections are a leading cause of death [2]. Most patients are followed with a “watch and wait” strategy for years until they meet criteria for therapy [3]. However, even in treatment-naïve patients, responses to pneumococcal, influenza, and hepatitis B vaccines are frequently impaired [4–8] and many require intravenous immunoglobulin (IVIg) infusions to mitigate infections [9]. The processes underlying the loss of immune function are still poorly understood.

Since their emergence hundreds of years ago [10], 4 human coronaviruses (HCoV-229E, HCoV-NL63, HCoV-HKU1, and HCoV-OC43), which cause mild seasonal upper respiratory infections [11], have become endemic. However, more recently there have been zoonotic outbreaks of 3 pathogenic HCoVs, including severe Acute Respiratory Syndrome Coronavirus (SARS-CoV), Middle East Respiratory Syndrome Coronavirus (MERS-CoV), and most recently SARS-CoV-2. The Coronavirus Disease of 2019 (COVID-19) caused by SARS-CoV-2 has resulted in the deaths of over 6 million people globally and over 1 million in the United States [12]. At the onset of the pandemic, severe illness and mortality were especially high in older individuals with comorbidities and compromised immunity [13,14]. Hence, SARS-CoV-2 has posed a particularly difficult challenge for CLL patients, especially before the availability of protective vaccines when COVID-19 fatality rates ranged from 27% to 38% [15,16]. Although mortality rates have since declined due to widespread vaccination campaigns [17], other mitigation strategies [18–21], and/or the evolution of less pathogenic variants [22], prevention of SARS-CoV-2 infection in CLL patients still remains a high priority.

SARS-CoV-2 enters human respiratory epithelial cells following the interaction of the receptor-binding domain (RBD) of the viral spike (S) glycoprotein with the host angiotensin-converting enzyme 2 (ACE2) receptor [23,24]. Neutralizing antibodies (NAbs) that disrupt RBD/ ACE2 binding, block viral entry and thus represent a key defense against SARS-CoV-2 infection and disease, as evidenced by the clinical benefits of convalescent plasma and monoclonal Abs capable of neutralizing sensitive SARS-CoV-2 strains [21,25,26]. NAbs are also known to prevent or mitigate SARS-CoV-2 disease [27,28]. Thus, NAbs represent an important correlate of immune protection [29].

Multiple reports have shown diminished immune responses in CLL patients following COVID-19 mRNA vaccination [30–33], with a recent review highlighting active treatment, hypogammaglobulinemia, and advanced age as the most common independent risk factors of poor vaccine outcomes [34]. However, few studies have focused specifically on treatment-naïve CLL patients and no study has dissected vaccine-induced responses to gauge the extent of adaptive immune dysfunction in CLL. Here, we performed a comprehensive analysis of both humoral and cellular immune responses in a clinically well-characterized cohort of SARS-CoV-2 infection-naïve CLL patients as well as healthy controls following 2 immunizations with either the Pfizer-BioNTech BNT162b2 or Moderna mRNA-1273 mRNA vaccines.

## Methods

### Ethics approval

We performed a prospective observational SARS-CoV-2 vaccination study at the University of Alabama at Birmingham in accordance with the principles of the Declaration of Helsinki and Good Clinical Principles guidelines. Written informed consent was obtained from all participants who were adults over age 18. The study was conducted following institutional review board (IRB) approval by the University of Alabama at Birmingham (IRB #130821005 and 160125005).

This study is reported as per the Strengthening the Reporting of Observational Studies in Epidemiology (STROBE) guideline (S1 STROBE Checklist).

### Study participants

We enrolled 95 CLL patients from outpatient clinics as well as 30 healthy controls, who were recruited on a rolling basis from the community. All participants received 2 doses of either the BNT162b2 or the mRNA-1273 vaccine, with the vaccine choice made by the participants (Table 1). Both vaccines encode the SARS-CoV-2 Wu-01 spike protein. Blood samples were collected within 3 months from the last immunization and processed to obtain peripheral blood mononuclear cells (PBMCs) and plasma as described [21,35]. None of the participants reported prior SARS-CoV-2 infection, which was confirmed by analyzing plasma samples for the presence of anti-SARS-CoV-2 nucleocapsid (N) antibodies using a commercially available (Abbott) test [36]. CLL patients were diagnosed according to the International Workshop on Chronic Lymphocytic Leukemia (IWCLL) guidelines [3], with clinical characteristics obtained by retrospective analysis of electronic medical records (Table 2 and S1 Table). These included demographics, disease and therapeutic history, Rai stage, absolute lymphocyte counts (ALCs), serum beta-2 microglobulin (β2M), immunoglobulin M (IgM), immunoglobulin A (IgA) and immunoglobulin G (IgG) levels, a requirement for IVIg prophylaxis, immunoglobulin heavy chain variable gene (*IGHV*) mutation status [37], expression of the surface marker CD38 (cluster of differentiation 38) (≥20%) [38], and cytogenetics analysis by fluorescence in situ hybridization (FISH) [39].

**Table 1:** Demographics of SARS-CoV-2 vaccinees.

Characteristic	Healthy controls (*n* = 30)	CLL (*n* = 95)
Age in years, median (IQR)	62 (44–70)	72 (64–77)
Age ≥65 years, number (%)	13 (43)	71 (75)
Sex, male, number (%)	16 (53)	47 (50)
**mRNA vaccine**		
Pfizer-BioNTech BNT162b2, number	27	61
Moderna mRNA-1273, number	3	34
Days from second vaccination to testing, median (IQR)	36 (28–57)	38 (27–83)

CLL, chronic lymphocytic leukemia; IQR, interquartile range; SARS-CoV-2, Severe Acute Respiratory Syndrome Coronavirus 2.

**Table 2:** Demographic and clinical characteristics of CLL patients by vaccine type.

	All CLL patients		Treatment-naïve CLL patients	
Patient characteristic	BNT162b2 (*n* = 61)	mRNA-1273 (*n* = 34)	BNT162b2 (*n* = 30)	mRNA-1273 (*n* = 15)
Age in years, median (IQR)	71 (64–78)	73 (66–76)	70 (61–77)	75 (65–78)
Age ≥65 years, number (%)	45 (74)	26 (77)	21 (70)	11 (73)
Sex, male, number (%)	28 (46)	19 (56)	14 (47)	8 (53)
Months from diagnosis to vaccination, median (IQR)	91 (54–152)	102 (33–162)	68 (34–96)	56 (22–141)
Days from second vaccination to testing, median (IQR)	38 (28–82)	38 (24–84)	56 (31–90)	65 (27–94)
**Rai stage** ^1^ **, number (%)**				
0–II	29/34 (85)	17/18 (94)	26/30 (87)	14/15 (93)
III–IV	5/34 (15)	1/18 (6)	4/30 (13)	1/15 (7)
**Disease/treatment status, number (%)**				
Treatment-naive	30 (49)	15 (44)	30 (100)	15 (100)
Active therapy	20 (33)	14 (41)	NA	NA
Off-therapy in remission	7 (12)	2 (6)	NA	NA
Off-therapy in relapse	4 (7)	3 (9)	NA	NA
**Molecular and phenotypic biomarkers, number (%)**				
*IGHV*, mutated	32/49 (65)	15/26 (58)	23/27 (85)	12/12 (100)
CD38 (≥20%)	13/57 (23)	10/33 (30)	8/27 (30)	3/15 (20)
**FISH, number (%)**				
Normal	5/59 (9)	5/31 (16)	5/29 (17)	2/13 (15)
del(13q)	33/59 (56)	15/31 (48)	16/29 (55)	10/13 (77)
Trisomy 12	12/59 (20)	3/31 (10)	5/29 (17)	0/13 (0)
del(11q)	6/59 (10)	4/31 (13)	2/29 (7)	0/13 (0)
del(17p)	3/59 (5)	4/31 (13)	1/29 (4)	1/13 (8)
**IVIg therapy**				
Number (%)	14/61 (23)	11/34 (32)	4/30 (13)	2/15 (13)
**Laboratory parameters, median (IQR)**				
Absolute lymphocyte count (10^9^/L)	8.4 (2.4–20)	4.9 (1.8–20)	13 (8.7–35)	13 (5.9–26)
β2-microglobulin (mg/L)	2.2 (1.9–3.0)	2.3 (1.7–3.2)	2.0 (1.6–2.4)	2.1 (1.7–2.9)
IgM (mg/dL)	25 (18–63)	29 (26–59)	28 (21–62)	29 (26–57)
IgG (mg/dL)	774 (561–961)	702 (559–936)	781 (611–981)	746 (695–1,240)
IgA (mg/dL)	114 (69–166)	87 (59–131)	103 (80–159)	122 (65–188)

^1^Rai stage was determined for treatment-naïve and patients off-therapy in relapse.

CD38, cluster of differentiation 38; CLL, chronic lymphocytic leukemia; del, deletion; FISH, fluorescence in situ hybridization; IgA, immunoglobulin A; IgG, immunoglobulin G; *IGHV*, immunoglobulin heavy chain variable gene; IgM, immunoglobulin M; IQR, interquartile range; IVIg, intravenous immunoglobulin; NA, not applicable.

### Serologic analyses

IgG-binding antibodies to the entire SARS-CoV-2 spike (S) protein, its RBD, as well as the S1 and S2 subunits, were detected by enzyme-linked immunosorbent assay (ELISA) using proteins derived from the Wu-01 strain [40–42]. Midpoint (EC_50_) and endpoint (EP) titers were determined as described [21,42]. Vaccinee plasma samples were also tested for IgG-binding antibodies to SARS-CoV, MERS-CoV, HCoV-HKU1, HCoV-OC43, HCoV-NL63, and HCoV-229E spike proteins by ELISA as described [21]. Assay sensitivity cut-off values for spike and RBD were >100 (for details see S1 Text).

### SARS-CoV-2 neutralization analyses

Plasma samples from vaccinees were tested for neutralizing responses against the SARS-CoV-2 variants D614G [43] and B.1.617.2 (also termed Delta) [44,45] using an HIV-1 based pseudovirus assay as described [46]. Briefly, pseudovirus stocks were generated by co-transfecting spike expression plasmids with an HIV-1 nanoluciferase encoding reporter backbone. Luciferase activity in wells with virus and no plasma were set to 100%, and the dilution of plasma at which luminescence was reduced to 50% (Inhibitory Dose 50; ID_50_) was calculated as an average of 2 technical replicates. Plasma from each vaccinee was analyzed on at least 2 occasions, with the geometric mean titer of all measurements reported. Values below a titer of 1:20 were treated as 20 when averaging. Plasma samples were also analyzed with an ACE2/RBD binding inhibition assay as described [21,47]. Assay sensitivity cut-off values for the D614G and Delta neutralization assays were >20 and for RBD/ACE2 binding >90% (for details see S1 Text).

### Analysis of cellular immune responses

PBMCs were immunophenotyped as described [35]. For activation-induced marker staining (AIM), PBMCs were stimulated with SARS-CoV-2 (Wu-01) derived N and S protein peptide pools (BEI Resources) in the presence of co-stimulatory anti-CD28 and anti-CD49d antibodies (BD Pharmingen). Cell aliquots from each sample were stimulated with dimethyl sulfoxide (DMSO) as a negative control and staphylococcal enterotoxin B (SEB) as a positive control. Intracellular staining (ICS) was performed in parallel with the AIM analyses [35], with events collected on a BD FACSymphony A3 instrument and analyzed using FlowJo software (v10). For both AIM and ICS analyses, responses were compared to an unstimulated control from the same participant. Responses were scored positive if they were at least 3 times higher than the unstimulated control as well as higher than the background of PBMCs that did not respond to antigen (using a Chi-square analysis with *p* < 0.05; S1 Text). Combinatorial polyfunctionality analysis of antigen-specific T-cell subsets (COMPASS) was calculated as described [48].

### Statistical analysis

Data were analyzed in R v4.0.5 and GraphPad Prism version 9.0 software. Associations between serologic or cellular responses with binary clinical data were analyzed using Fisher’s exact test and with continuous clinical variables using the Mann–Whitney test. Calculations of *p*-values were adjusted for multiple comparisons of serologic, cellular, and clinical data using the Benjamini–Hochberg procedure for false discovery rate. For comparisons of more than 2 categories, *p*-values were determined by Dunn’s test of multiple comparisons following a Kruskal–Wallis test. Firth logistic regression was used to examine the association of serologic or cellular responses with clinical variables. GraphPad Prism version 9.0 software was used to plot these analyses. Significance was determined as a *p*-value <0.05 unless otherwise stated.

## Results

Ninety-five patients diagnosed with CLL according to IWCLL criteria [3] and 30 healthy controls were enrolled into an observational vaccine cohort. None of the participants had evidence for prior SARS-CoV-2 infection as determined by clinical history and a lack of nucleocapsid antibodies. All participants were immunized between December 2020 and June 2021 and received 2 doses of either the Pfizer BNT162b2 or the Moderna mRNA-1273 vaccine. This period coincided with the spread of the SARS-CoV-2 D614G and Delta variants in the United States. The median age of the CLL patients was 72 years (IQR, 64 to 77) and 47 (50%) were male (Table 1). Forty-five of the patients (47%) were treatment-naïve, whereas 50 (53%) had prior therapy, including 34 who were being actively treated (i.e., anti-CD20 therapy, Bruton’s tyrosine kinase [BTK] inhibition) (Table 2 and S1 Table). Seven individuals were refractory to therapy and relapsed, while 9 were off therapy in clinical remission. Sixty-one patients (64%) received 30 μg of the Pfizer-BioNTech BNT162b2 vaccine, while 34 (36%) were immunized with 100 μg of the Moderna mRNA-1273 vaccine according to FDA guidelines. The median time from the second immunization to testing was 38 days (IQR, 27 to 83) for CLL patients and 36 days (IQR, 28 to 57) for healthy controls (Table 1).

### Binding and neutralizing antibody responses in CLL patients correlate with disease status

**Fig 1:**
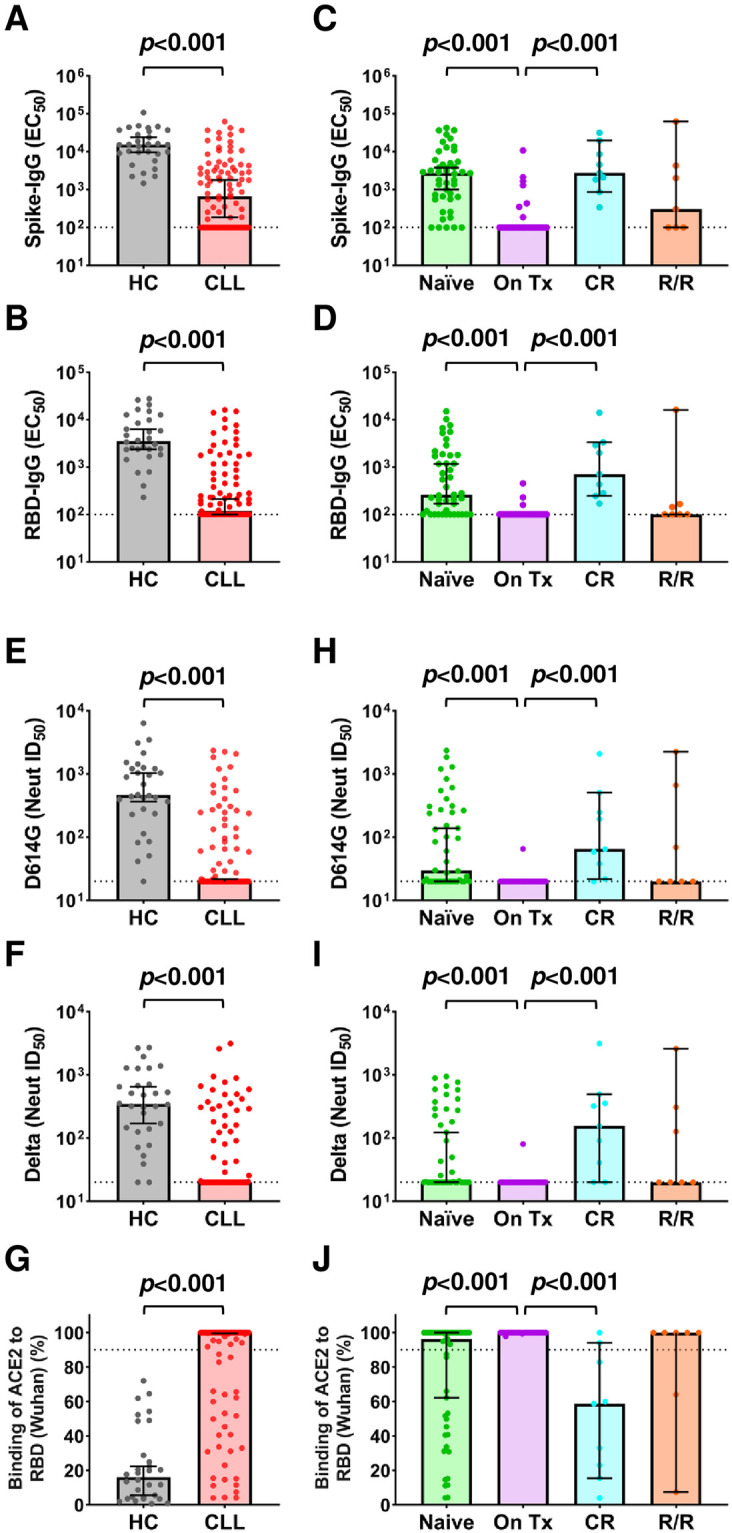
Serologic responses in SARS-CoV-2 mRNA vaccinated CLL patients and HC. (A and B) SARS-CoV-2 vaccine-elicited anti-spike (A) and anti-RBD (B) IgG titers expressed as half-maximal effective concentrations (EC_50_). (C and D) Spike (C) and RBD (D) IgG titers stratified by CLL disease status: treatment-naïve (Naïve), on-therapy (On Tx), off therapy in CR, and off therapy and relapsed or refractory (R/R). See Tables 1 and 2 for demographic and clinical details of participants. (E–G) Vaccine-elicited NAb titers against the (E) D614G and (F) Delta SARS-CoV-2 variants expressed as the reciprocal half-maximal inhibitory dilution (ID_50_) or by (G) ACE2/RBD (Wu-01 strain) binding inhibition at a 1:25 dilution. (H–J) NAb titers for D614G (H) and Delta (I) variants and ACE2/RBD binding frequencies (J) stratified by CLL disease status. Bars indicate the median with 95% CI. Dotted black lines indicate assay sensitivity cutoffs (EC_50_ values of <100 by ELISA, ID_50_ values of <20 in neutralization assays, and >90% ACE2 binding in the RBD-inhibition assay). Calculations of *p*-values were determined by the Mann–Whitney test (A, B, E–G) or Dunn’s test of multiple comparisons following a Kruskal–Wallis test (C, D, H–J). ACE2, angiotensin-converting enzyme 2; CI, confidence intervals; CLL, chronic lymphocytic leukemia; CR, clinical remission; ELISA, enzyme-linked immunosorbent assay; HC, healthy control; IgG, immunoglobulin G; NAb, neutralizing antibody; RBD, receptor-binding domain; SARS-CoV-2, Severe Acute Respiratory Syndrome Coronavirus 2.

Plasma samples from all vaccinees were tested for seroreactivity to the SARS-CoV-2 spike (S) and RBD antigens by ELISA [21,42]. While all control participants generated both anti-S and anti-RBD IgG following immunization, response rates were reduced in CLL patients (both *p* < 0.001), with only 65 (68%) developing anti-S and 51 (54%) developing anti-RBD antibodies (S2–S4 Tables). CLL vaccinees also had 23-fold lower anti-S and 30-fold lower anti-RBD half-maximal effective concentrations (EC_50_) compared to healthy controls (both *p* < 0.001; Fig 1A and 1B). Even when comparing only CLL patients who mounted a humoral response (i.e., CLL responders), we found median IgG anti-S and anti-RBD EC_50_ values to be 7.2-fold and 6.4-fold lower than those of healthy controls, respectively (S2 and S3 Tables). Importantly, age did not explain the observed differences between CLL patients and healthy controls, who had a median age 10 years younger (Table 1). A comparison of serologic responses in healthy control participants stratified by age (older or younger than 65 years) with CLL responders yielded the same results (*p* < 0.001; S1 Fig).

We next explored whether there was an association between the humoral response and disease history. As expected, treatment-naïve patients had higher response rates and anti-S Ab titers (median EC_50_ 2,733) compared to vaccinees on active CLL therapy (median EC_50_ <100), many of whom failed to seroconvert (*p* < 0.001; Fig 1C, S3 and S5 Tables). All CLL patients who were in clinical remission (CR) mounted anti-S and anti-RBD responses and had significantly higher IgG titers (median EC_50_ 2,740 and 708) compared to those on treatment (*p* < 0.001; Fig 1C and 1D, S3 and S5 Tables). RBD response rates and Ab titers were also higher for individuals who were treatment-naive or in clinical remission compared to actively treated patients (both *p* < 0.001), and vaccinees who were refractory to therapy or relapsed (R/R) had generally low anti-S and anti-RBD responses (Fig 1C and 1D). Although most individuals who were treatment-naive or in clinical remission had anti-S and anti-RBD binding Abs, their titers were significantly lower compared to healthy controls (*p* ≤ 0.02; Fig 1A–1D, S3 and S4 Tables).

We next analyzed plasma samples for neutralizing activity against the SARS-CoV-2 D614G [43] and Delta variants [44,45] using an HIV-1-based pseudovirus assay [46]. Consistent with the ELISA findings, neutralizing responses in CLL patients were overall reduced compared to healthy controls (both *p* < 0.001; Fig 1E and 1F, S2–S4 Tables). While NAbs against the D614G and Delta variants were found in 97% and 93% of healthy controls, respectively, response rates in CLL patients were significantly lower at 42% (40/95) and 38% (35/93) (*p* < 0.001; S2–S4 Tables). An assay that measured Ab-mediated inhibition of the ACE2/RBD (Wu-01 strain) interaction [21], yielded very similar results, detecting blockade in all controls, but in only 30% (28/95) of CLL patients (*p* < 0.001; S2–S4 Tables). Median NAb titers were also significantly lower in CLL patients than healthy controls, with half-maximal inhibitory dilutions (ID_50_) for D614G being >23-fold (464 versus ≤20; *p* < 0.001; Fig 1E) and Delta being >17-fold (346 versus ≤20; *p* < 0.001; Fig 1F) lower, respectively. Similarly, ACE2/RBD inhibition was lower in CLL patients (*p* < 0.001; Fig 1G). Finally, neutralizing responses in CLL patients reflected their disease and treatment status. Response rates, NAb titers, and ACE2/RBD blockade were all significantly higher in individuals who were treatment-naive or in clinical remission compared to individuals on active treatment (*p* < 0.001; Fig 1H–1J, S5 Table). These data confirm and extend earlier findings [30,31,49], showing impaired humoral responses not only in actively treated but also in treatment-naïve CLL patients.

### BNT162b2 vaccination is a negative predictor of SARS-CoV-2 NAb elicitation in CLL

To search for predictors of humoral responses following immunization, we analyzed the demographics and disease characteristics of CLL vaccinees. By univariate analysis, we compared 18 clinical variables (listed in S1 Table) with binding (anti-S and anti-RBD IgG) and neutralizing (D614G, Delta, and ACE2/RBD blockade) antibody responses, measured as binary outcomes (Tables 3 and 4). Cut-off values for S and RBD binding were >100, for D614G and Delta neutralization >20, and >90% for RBD/ACE2 blockade. Examining the 4 CLL groups (treatment-naive, active therapy, clinical remission, and refractory/relapsed), disease status itself was significantly associated with humoral responsivity (all *p* < 0.001; Table 3). Clinical determinants that were associated with higher binding and neutralizing Ab titers included early Rai stage disease (0–II), low serum β2-microglobulin (≤2.4 mg/L) levels, lack of prior CLL therapy, vaccination ≥12 months following anti-CD20 therapy, and no requirement for IVIg therapy (all *p* ≤ 0.04; Table 3). Since D614G and Delta NAb titers were very similar, we performed a multivariate analysis using only the D614G NAb data to determine clinical risk factors associated with a failure to mount a serological response (Table 4). As expected, active therapy was a significant adverse predictor of both anti-S binding (OR 62, 95% CI 3.6 to 3,500, *p* = 0.003) and D614G NAbs (OR 40, 95% CI 1.2 to 2,500, *p* = 0.04), whereas being refractory to therapy in relapse (OR 7.7, 95% CI 1.1 to 64, *p* = 0.04) and requiring prophylactic IVIg therapy (OR 5.2, 95% CI 1.3 to 29, *p* = 0.02) were both associated with poor binding Ab responses. Unexpectedly, BNT162b2 vaccination was also a negative predictor of D614G NAbs (OR 5.8, 95% CI 1.6 to 27, *p* = 0.006), suggesting vaccine-specific differences in NAb elicitation.

**Table 3:** Clinical predictors of humoral immune responses in CLL vaccinees determined by univariate analysis.

Clinical variable		Spike (EP)	RBD (EP)	D614G (Neut ID_50_)	Delta (Neut ID_50_)	ACE2/RBD Binding
Age (< or ≥65 years)	*p*-value OR 95% CI	0.8 1.1 0.4, 3.3	1 1.0 0.3, 2.7	1 1 0.4, 3	1 1 0.4, 3	0.6 1.3 0.4, 3.8
Sex (male vs. female)	*p*-value OR 95% CI	0.8 1.2 0.5, 3.1	0.7 0.8 0.3, 2	0.8 0.9 0.4, 2.1	0.8 0.9 0.4, 2.2	0.7 0.8 0.3, 2.1
Disease/treatment status^1^	*p*-value OR 95% CI	**<0.001** NA NA	**<0.001** NA NA	**<0.001** NA NA	**<0.001** NA NA	**<0.001** NA NA
Rai stage (0–II vs. III–IV)	*p*-value OR 95% CI	0.5 0.5 0.04, 28	**0.04** **0.2** **0.01, 1.2**	**0.03** **0.1** **0.002, 1.1**	0.09 0.2 0.003, 1.6	0.4 3.8 0.38, 191
*IGHV* (mutated vs. unmutated)	*p*-value OR 95% CI	**0.02** **0.3** **0.1, 0.9**	**0.01** **0.3** **0.1, 0.8**	0.1 0.5 0.2, 1.3	0.3 0.6 0.2, 1.5	0.07 2.5 0.9, 7
CD38 (< or ≥20%)	*p*-value OR 95% CI	1 0.9 0.3, 2.9	0.2 0.5 0.2, 1.4	0.8 0.8 0.3, 2.4	1 1.1 0.4, 3.2	0.8 1.3 0.4, 4.6
FISH cytogenetics^2^	*p*-value OR 95% CI	0.5 NA NA	0.08 NA NA	0.6 NA NA	0.4 NA NA	0.1 NA NA
β2M (≤ vs. >2.4 mg/dL)	*p*-value OR 95% CI	**0.003** **4.3** **1.5, 13**	**0.02** **3** **1.1, 8.7**	**<0.001** **5.8** **1.9, 20**	**0.009** **3.9** **1.3, 14**	**0.01** **0.2** **0.05, 0.8**
Any prior therapy (yes or no)	*p*-value OR 95% CI	**<0.001** **0.04** **0.004, 0.2**	**<0.001** **0.1** **0.05, 0.4**	**<0.001** **0.2** **0.07, 0.5**	**0.003** **0.3** **0.1, 0.7**	**0.01** **3.3** **1.2, 9.6**
Anti-CD20 (< vs. ≥12 months)	*p*-value OR 95% CI	**0.008** **8.4** **1.4, 92**	**0.001** **Inf** **2.7, Inf**	**0.003** **Inf** **2, Inf**	**0.007** **Inf** **1.6, Inf**	**0.02** **0** **0, 0.9**
BTK inhibitor therapy (yes or no)	*p*-value OR 95% CI	1 0.9 0.09, 7.3	1 1.2 0.01, 104	1 0 0, Inf	1 0 0, Inf	1 0 0, Inf
ALC (< vs. ≥5.0 × 10^9^/L)	*p*-value OR 95% CI	**0.03** **2.9** **1.1, 8.1**	**0.01** **2.9** **1.2, 7.3**	0.2 1.7 0.7, 4.3	0.4 1.6 0.6, 4.1	0.1 0.5 0.2, 1.3
IVIg therapy (yes or no)	*p*-value OR 95% CI	**0.005** **0.2** **0.08, 0.7**	**0.02** **0.3** **0.1, 0.9**	**0.01** **0.3** **0.07, 0.8**	0.05 0.3 0.08, 1	0.1 2.7 0.8, 12
IgM (< vs. ≥40 mg/dL)	*p*-value OR 95% CI	0.1 2.9 0.7, 18	0.5 1.5 0.5, 5	0.3 1.9 0.6, 5.7	0.6 1.4 0.5, 4.4	0.1 0.4 0.1, 1.2
IgG (< vs. ≥650 mg/dL)	*p*-value OR 95% CI	**0.01** **4.7** **1.3, 20**	**0.003** **4.8** **1.6, 16**	0.09 2.6 0.9, 8.1	0.2 2.1 0.7, 6.6	0.2 0.5 0.1, 1.6
IgA (< vs. ≥60 mg/dL)	*p*-value OR 95% CI	0.07 3.4 0.8, 14	**0.04** **3.7** **1.0, 16**	0.4 2.1 0.5, 8.9	0.6 1.5 0.4, 6.7	0.8 0.7 0.2, 2.9
Pfizer vs. Moderna vaccine	*p*-value OR 95% CI	1 1.1 0.4, 2.8	0.5 0.7 0.3, 1.8	0.1 0.5 0.2, 1.3	0.1 0.5 0.12, 1.3	0.1 2.3 0.9, 6.4
Months from vaccination^3^	*p*-value OR 95% CI	0.1 NA NA	0.05 NA NA	0.3 NA NA	0.9 NA NA	1 NA NA

^1^Disease/treatment status includes patients who are treatment-naïve, on active therapy, off-therapy in remission and off-therapy in relapse.

^2^FISH cytogenetics includes normal, del(13q), trisomy 12, del(11q), and del(17p) status.

^3^Patients were categorized as ≤1, 2, or ≥3 months from second vaccination.

Assay sensitivity cut-off values for Spike and RBD were >100; for the D614G and Delta neutralization assays >20; and >90% for RBD/ACE2 binding.

Binary outcomes with significant *p*-values are in bold.

ACE2, angiotensin-converting enzyme-2; ALC, absolute lymphocyte count; β2M, beta-2 microglobulin; BTK, Bruton’s tyrosine kinase; CD20, cluster of differentiation 20; CD38, cluster of differentiation 38; CI, confidence interval; CLL, chronic lymphocytic leukemia; del, deletion; EP, endpoint titer; FISH, fluorescence in situ hybridization; IgA, immunoglobulin A; IgG, immunoglobulin G; IgM, immunoglobulin M; IVIg, intravenous immunoglobulin; Inf, infinity; *IGHV*, immunoglobulin heavy chain variable region gene; Moderna, mRNA-1273; NA, not applicable; Neut ID_50_, half-maximal neutralizing titer; OR, odds ratio; Pfizer-BioNTech, BNT162b2; RBD, receptor binding domain.

**Table 4:** Multivariate analysis of humoral immune responses with clinical variables in CLL vaccinees.

Clinical variable	Spike (EP)			D614G (Neut ID_50_)		
	*p*-value	OR	95% CI	*p*-value	OR	95% CI
Age ≥65 years	0.3	0.4	0.08, 1.9	0.9	1.1	0.3, 4.5
Sex, male	0.7	1.4	0.4, 5.6	0.7	1.3	0.4, 4.4
Rai stage III–IV	0.6	1.8	0.1, 18	0.2	3.5	0.5, 56
*IGHV*, unmutated	0.9	0.9	0.1, 6	0.8	1.2	0.3, 5.1
Active therapy	**0.003**	**62**	**3.6, 3,500**	**0.04**	**40**	**1.2, 2,500**
Off-therapy in remission	0.5	3.7	0.02, 250	0.8	0.7	0.03, 17
Off-therapy in relapse	**0.04**	**7.7**	**1.1, 64**	0.6	1.7	0.3, 13
ALC ≥5.0 × 10^9^/L	0.3	3.8	0.4, 130	0.1	5.2	0.7, 110
IVIg therapy requirement	**0.02**	**5.2**	**1.3, 29**	0.05	4.4	1.0, 25
Pfizer BNT162b2 vaccination	0.6	1.4	0.4, 6	**0.006**	**5.8**	**1.6, 27**
2 months from vaccination	0.3	2.3	0.5, 12	0.9	1.1	0.2, 5
≥3 months from vaccination	0.5	0.6	0.1, 2.8	0.9	1.1	0.3, 5

Significant *p*-values are in bold.

ALC, absolute lymphocyte count; CI, confidence interval; CLL, chronic lymphocytic leukemia; EP, endpoint titer; *IGHV*, immunoglobulin heavy chain variable region gene; IVIg, intravenous immunoglobulin; Neut ID_50_, half-maximal neutralizing titer; OR, odds ratio.

### Vaccinated CLL patients have reduced CD4^+^ but relatively preserved CD8^+^ T cell functions

To investigate the impact of vaccination on cell-mediated immunity, we examined PBMCs from a representative subset of vaccinated CLL patients (*n* = 36) and healthy controls (*n* = 21) for which sufficient samples were available (S6 Table). The frequencies of circulating CD3 (cluster of differentiation 3), CD4 (cluster of differentiation 4), and CD8 (cluster of differentiation 8) positive T cells as well as naïve and memory subsets were determined by multicolor flow cytometry analysis using CD45RA and CCR7 immunophenotyping (S2 Fig). As expected, total CD3^+^ T cell frequencies were significantly higher in controls than CLL vaccinees (*p* < 0.001; S2A Fig, S7A Table). Immunophenotypic analysis showed skewing of the CLL T cell compartment, with lower total CD4^+^ (*p* = 0.01) and higher CD8^+^ (*p* = 0.002) T cell numbers, resulting in lower CD4:CD8 ratios (*p* = 0.004) compared to controls (S2A Fig). Among T cell subsets, CLL vaccinees had lower frequencies of naïve CD4^+^ and CD8^+^ T cells (T_N_; *p* = 0.02 and *p* < 0.001, respectively; S1B and S1C Fig). In contrast, CLL effector memory (T_EM_) CD8^+^ (*p* = 0.004), but not CD4^+^, T cell frequencies were higher. However, central memory (T_CM_) and terminally differentiated effector memory (T_EMRA_) T cells did not differ between the groups (S1B and S1C Fig). These results confirmed previous findings [50–53], indicating lower naïve CD4^+^ and CD8^+^ T cell populations as well as higher effector memory CD8^+^ T cells in CLL vaccinees compared to healthy controls.

**Fig 2:**
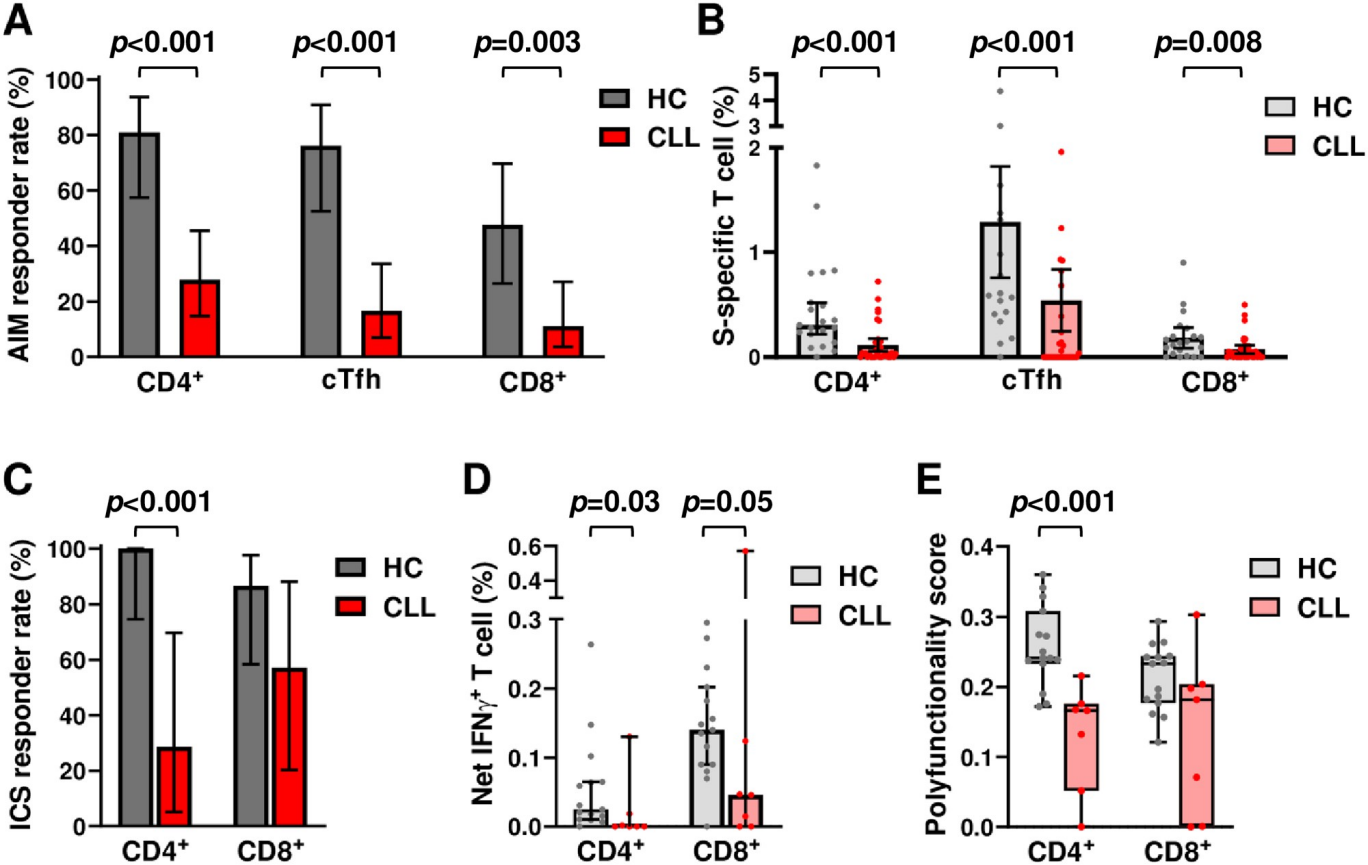
T cell subset responses and effector function in SARS-CoV-2 mRNA vaccinees. (A) S-specific T cell AIM response rates among CD4^+^, cTfh, and CD8^+^ T cell subpopulations in SARS-CoV-2 mRNA vaccinated HC (gray) and CLL (red) participants. (B) Quantitative comparisons of S-specific T cell AIM response frequencies for CD4^+^, cTfh, and CD8^+^ T cells in HC and CLL vaccinees. (C) ICS response rates of S-specific CD4^+^ and CD8^+^ T cells from HC and CLL vaccinees. Responders were defined as individuals with reactivity against at least 1 of 5 effector features (S1 Text, S7D Table) upon peptide stimulation. (D) Quantitative comparisons of IFNγ production by CD4^+^ and CD8^+^ T cells. (E) Comparisons of CD4^+^ and CD8^+^ T cell effector responses calculated using a combinatorial polyfunctionality analysis [48]. Bars indicate the (A and C) mean or (B, D, E) median with 95% CI. Calculations of *p*-values were determined by Fisher’s exact test (A and C) or the Mann–Whitney test (B, D, E). AIM; activation-induced marker; CD4, cluster of differentiation 4; CD8, cluster of differentiation 8; CI, confidence interval; CLL, chronic lymphocytic leukemia; cTfh, circulating T follicular helper; HC, healthy control; ICS, intracellular staining; IFNγ, interferon gamma; SARS-CoV-2, Severe Acute Respiratory Syndrome Coronavirus 2; S, spike.

To examine antigen-specific function, we determined activation-induced marker (AIM) expression for CD4^+^, circulating T follicular helper (cTfh), and CD8^+^ cells following S and N peptide pool (Wu-01 strain) stimulation. For both CD4^+^ and cTfh cells, antigen specificity was quantified by the frequency of PD-L1 and OX40 co-expressing cells, while the CD69^+^CD137^+^ population was used to identify reactive CD8^+^ T cells (S3A Fig). Although all participants lacked N antigen seroreactivity, AIM T cell responses against N peptides were detected in the CD8^+^ T cells of 1 healthy control and the CD4^+^ T cells of 3 CLL patients (S7B Table), likely representing responses to prior endemic HCoV infections [54,55]. In contrast, overall responses against S peptides were found in 91% of controls, but only 33% of CLL patients (OR 0.05; 95% CI 0.01 to 0.27, *p* < 0.001; S7C Table). S-restricted responder rates for CLL vaccinees were also significantly lower for each of the 3 T cell subsets analyzed (all *p* ≤ 0.003; Fig 2A). Moreover, the median frequencies of S reactive AIM responding cells among the 3 T cell subsets were significantly reduced in CLL vaccinees compared to healthy controls (all *p* ≤ 0.008; Fig 2B). These findings demonstrate lower antigen-specific responses by different T cell subsets in CLL patients following SARS-CoV-2 mRNA vaccination.

We next tested CD4^+^ and CD8^+^ T cell function by quantifying cytokine and effector molecule production by ICS. To exclude potential effects of prior HCoV infections, only healthy controls (*n* = 15) and CLL (*n* = 7) samples with positive S and negative N peptide responses were analyzed. ICS positivity was defined by a T cell response to at least one of 5 parameters: IFNγ, IL-2, TNFα, CD107a plus granzyme B, or CD107a plus perforin (S3B Fig). S-restricted CD4^+^ T cell responses were significantly higher in controls compared to CLL vaccinees (*p* < 0.001; Fig 2C, S7D Table), with most pronounced differences observed for IFNγ (*p* = 0.03; Fig 2D). By combinatorial polyfunctionality analysis [48], a higher score for CD4^+^ T cells indicated more robust effector function for this subset in healthy controls compared to CLL vaccinees (*p* = 0.001; Fig 2E). In contrast, the responder rate and single or polyfunctionality quantitation for CD8^+^ T cells was comparable between the cohorts, although CLL vaccinees showed a trend toward lower IFNγ production. The difference between CD4^+^ and CD8^+^ T cell function was generally consistent between the AIM and ICS analyses. Univariate analyses to define potential clinical correlates with these T cell studies did not identify significant associations, likely reflecting low response rates, a small sample size, and/or the testing of only a subset of the total cohort. Overall, these data indicate reduced S-restricted CD4^+^ T cell effector functions, but relatively preserved CD8^+^ T cell functions in CLL vaccinees.

### Vaccinees who lack SARS-CoV-2 NAbs maintain Abs to endemic coronaviruses

**Fig 3:**
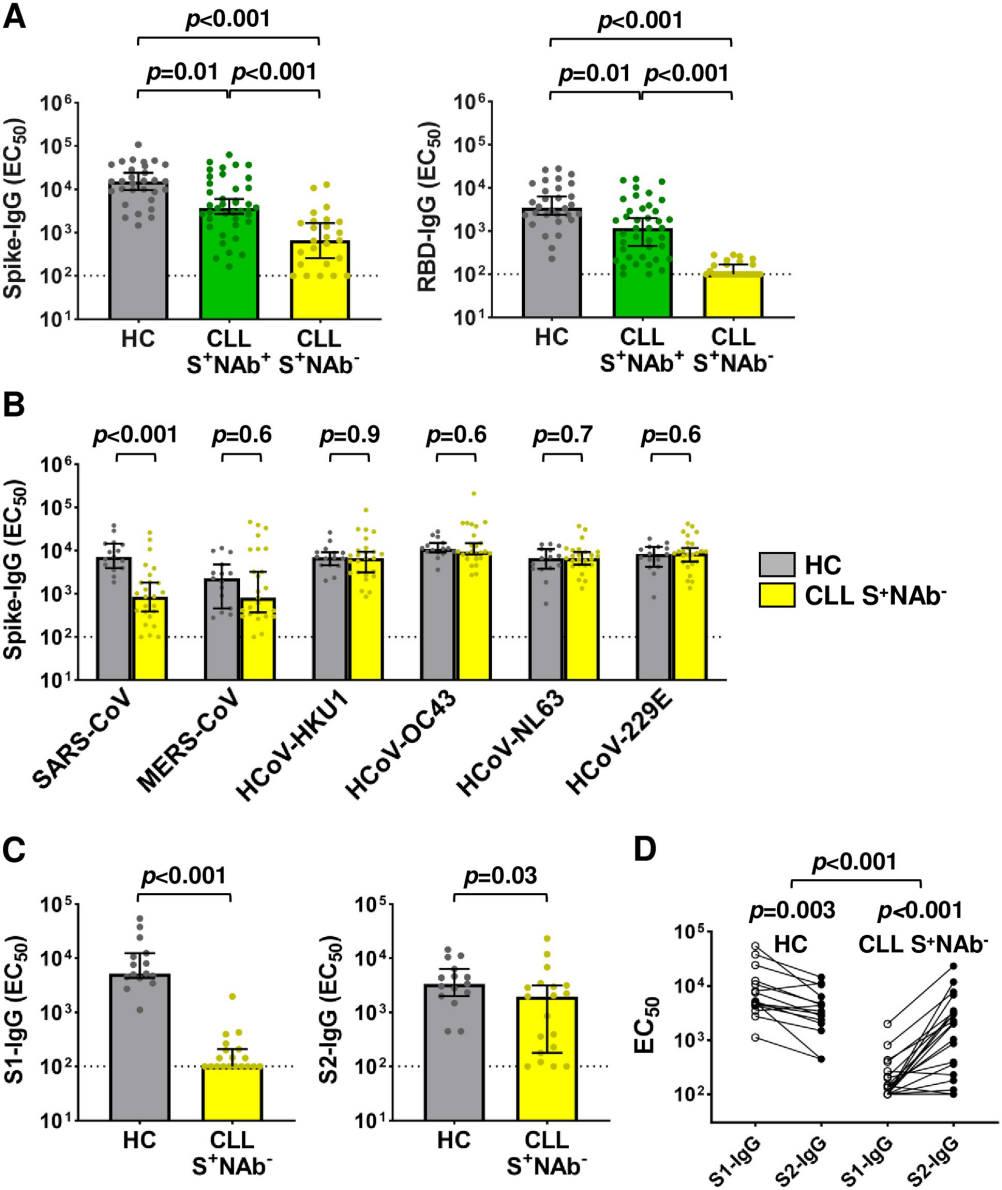
SARS-CoV-2 CLL vaccinees lacking NAbs maintain antibody responses to endemic HCoVs that cross-react with SARS-CoV and MERS-CoV. (A) IgG-binding antibody titers (EC_50_) against SARS-CoV-2 full-length S and RBD proteins for HC (*n* = 30), S^+^NAb^+^ CLL (*n* = 40), and S^+^NAb^-^ CLL (*n* = 25) vaccinees. (B) IgG-binding antibody (EC_50_) titers against the S proteins of 2 pathogenic and 4 endemic HCoVs from SARS-CoV-2 vaccinated HC (*n* = 15) and CLL S^+^NAb^-^ (*n* = 25) samples. (C) IgG-binding antibody (EC_50_) titers against the S1 and S2 subunits of the SARS-CoV-2 spike in HC (*n* = 15) and S^+^NAb^-^ CLL (*n* = 19) vaccinees. (D) Paired comparisons of S1 vs. S2 EC_50_ titers for the HC and S^+^NAb^-^ CLL samples analyzed in (C). Bars indicate the median with 95% CI. Dotted black lines indicate assay sensitivity cutoffs (EC_50_ values of <100). Calculations of *p*-values were determined by Dunn’s test of multiple comparisons following a Kruskal–Wallis test (A), the Mann–Whitney test (B and C), and paired Mann–Whitney tests for comparisons of S1 and S2 differences within individuals and an unpaired Mann–Whitney test on S1 and S2 differences to compare between cohorts (D). CI, confidence interval; CLL, chronic lymphocytic leukemia; EC_50_, half-maximal effective concentration; HC, healthy control; HCoV, human coronavirus; IgG, immunoglobulin G; MERS-CoV, Middle East Respiratory Syndrome Coronavirus; NAb, neutralizing antibody; RBD, receptor binding domain; S1 and S2, spike protein subunits 1 and 2; SARS-CoV-2, Severe Acute Respiratory Syndrome Coronavirus 2; S, spike.

An analysis of the serologic data (S2 and S3 Tables) showed that 32% of all CLL patients failed to seroconvert or develop D614G NAbs following vaccination (S^-^NAb^-^), while 42% developed both anti-S binding Abs and NAbs (S^+^NAb^+^). Unexpectedly, 26% of CLL vaccinees had anti-S binding Abs, but lacked detectable NAbs (S^+^NAb^-^). This was not due to IVIg therapy, since among the 25 IVIg treated CLL patients 14 were S^-^NAb^-^ and 6 were S^+^NAb^-^ (S1 and S2 Tables). Instead, the data suggested that some CLL patients had selectively lost their ability to generate NAbs despite exhibiting anti-S reactivity. To examine this unusual phenotype, we compared anti-S and anti-RBD binding Ab titers in S^+^NAb^+^ and S^+^NAb^-^ CLL patients. Median IgG titers against these 2 antigens were 3- to 4-fold lower for S^+^NAb^+^ CLL patients compared to healthy controls (*p* = 0.01) but were even more diminished for individuals with S^+^NAb^-^ status (*p* < 0.001), i.e., 23-fold and 35-fold, respectively (Fig 3A).

One potential explanation for the S^+^NAb^-^ serologic phenotype was the presence of cross-reactive Abs from prior HCoV infections. To investigate this possibility, we tested all S^+^NAb^-^ CLL patients (*n* = 25) and a subset of the healthy controls (*n* = 15) for IgG-binding Abs to recombinant spike proteins of 6 HCoVs: SARS-CoV, MERS-CoV, HCoV-HKU1, HCoV-OC43, HCoV-NL63, and HCoV-229E. We found that IgG EC_50_ titers against the SARS-CoV spike, which shares approximately 75% ectodomain sequence identity with SARS-CoV-2 [23,24], were significantly lower for S^+^NAb^-^ CLL patients than controls (*p* < 0.001), and a similar trend was observed for the more distantly related MERS-CoV spike. However, no such differences were found for the other HCoV spike proteins, against which high-titer Abs were detected in both CLL vaccinees and healthy controls (Fig 3B). Thus, while S^+^NAb^-^ vaccinees were unable to produce SARS-CoV-2–specific NAbs, they maintained high-titer HCoV-specific Abs. Moreover, these HCoV-specific Abs cross-reacted with the spike proteins of SARS-CoV and MERS-CoV since none of our participants had prior exposure to these viruses.

To further dissect the S^+^NAb^-^ serotype, we analyzed available samples from S^+^NAb^-^ CLL vaccinees (*n* = 19) and healthy controls (*n* = 15) for IgG binding against the SARS-CoV-2 S1 and S2 subunits by ELISA (S2 Table). CLL EC_50_ titers against these 2 S protein regions were again significantly lower compared to controls (*p* < 0.001 and *p* = 0.03); however, this difference was much more pronounced for S1 (52-fold) than S2 (1.7-fold) (Fig 3C). Moreover, a paired donor analysis of S1 and S2-IgG EC_50_ titers (Fig 3D) revealed that S^+^NAb^-^ CLL vaccinees had higher S2 titers than the controls (*p* = 0.003) who had elevated S1 titers (*p* < 0.001). This S2 bias remained significant even when median differences in S2-S1 titers were subtracted (*p* < 0.001). These results indicated the maintenance of preexisting HCoV antibodies that cross-reacted with conserved epitopes in the SARS-CoV and MERS-CoV spike proteins.

### mRNA-1273 elicits superior NAb responses in treatment-naive CLL patients

**Fig 4:**
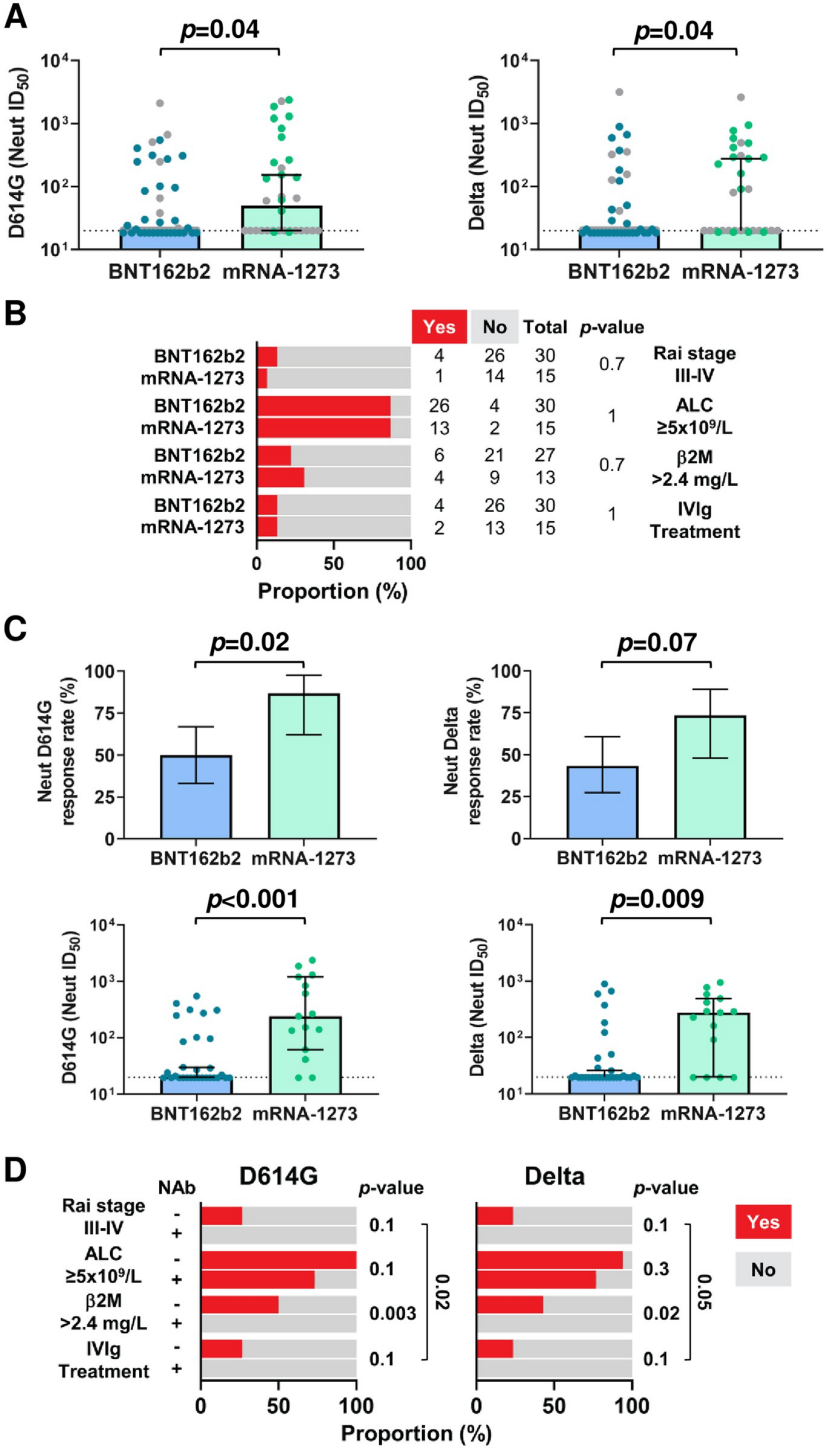
mRNA-1273 elicits higher NAb responses in treatment-naïve CLL vaccinees. (A) ID_50_ neutralizing titers against the SARS-CoV-2 D614G and Delta variants in all CLL patients who received either the BNT162b2 or the mRNA-1273 vaccine. Treatment-naïve BNT162b2 (blue) or mRNA-1273 (green) CLL vaccinees are color-coded relative to patients with all other disease states (gray). (B) Frequencies of clinical features including Rai stage III–IV, ALC (≥5 × 10^9^/L), serum beta-2 microglobulin (β2M; >2.4 mg/L), and IVIg prophylaxis therapy, in treatment-naive CLL patients by vaccine type. (C) Response rates and neutralization titers against the D614G and Delta variants in treatment-naive CLL patients by vaccine type. (D) Clinical features in treatment-naive BNT162b2 CLL vaccinees stratified by neutralization status against the SARS-CoV-2 D614G (NAb^+^ [*n* = 15] or NAb^-^ [*n* = 15]) and Delta (NAb^+^ [*n* = 13] or NAb^-^ [*n* = 17]) variants. Bars indicate the mean (upper plots in C) or median with 95% CI (A and lower plots in C). Calculations of *p*-values were determined with Fisher’s exact test (B, upper plots in C, and D) or the Mann–Whitney test (A and lower plots in C). ALC, absolute lymphocyte count; CI, confidence interval; CLL, chronic lymphocytic leukemia; ID_50_, reciprocal half-maximal inhibitory dilution; IVIg, intravenous immunoglobulin; NAb, neutralizing antibody; SARS-CoV-2, Severe Acute Respiratory Syndrome Coronavirus 2.

A multivariate analysis identified significantly higher D614G NAb response rates in CLL patients vaccinated with mRNA-1273 (53%, 18/34) compared to those vaccinated with BNT162b2 (36%, 22/61) (OR 5.8, 95% CI 1.6 to 27, *p* = 0.006; Table 4), despite very similar treatment and clinical states (Table 2). To examine the reason for this difference, we compared NAb ID_50_ titers in all patients by vaccine type. Remarkably, both the median D614G and Delta ID_50_ NAb titers of mRNA-1273 immunized CLL patients were significantly higher than those of CLL vaccinees who received BNT162b2 (both *p* = 0.04; Fig 4A). Since a large number of patients was unable to mount a humoral response because of advanced disease and/or immunosuppressive therapy (gray symbols in Fig 4A, Table 2), we next focused on treatment-naïve BNT162b2 (*n* = 30) and mRNA-1273 (*n* = 15) CLL vaccinees. Although only 28 of 45 treatment-naïve CLL vaccinees developed D614G NAbs, the response rates were significantly higher for mRNA-1273 (13/15, 87%) than for BNT162b2 (15/30, 50%) recipients (Fig 4C). In fact, mRNA-1273 vaccinees had 1.7-fold higher odds (OR 6.5, 95% CI 1.3 to 32, *p* = 0.02) of NAb development than BNT162b2 recipients. Similar results were obtained for the Delta variant, where 11 of 15 (73%) mRNA-1273 vaccinees mounted detectable NAbs compared to 13 of 30 (43%) BNT162b2 recipients, although these differences did not reach significance (Fig 4C). Finally, both D614G (*p* < 0.001) and Delta (*p* = 0.009) NAb titers were significantly higher in mRNA-1273 compared to BNT162b2 recipients (Fig 4C), despite very similar clinical characteristics (Table 2). Since these 2 groups had very similar Rai stages, ALCs, serum β2M levels, and IVIg prophylaxis requirements (Fig 4B and Table 2), these data indicated that the inferior NAb responses in CLL patients immunized with BNT162b2 were not a consequence of differences in disease progression.

Among the treatment-naïve CLL patients who received the BNT162b2 vaccine, half developed D614G NAbs (*n* = 15), while the other half did not (*n* = 15), thus providing an opportunity to examine possible reasons for these differences. The median time from the second vaccination to sample collection did not differ significantly between the NAb^+^ and NAb^-^ groups (Table 5). However, comparisons of clinical features indicated a trend toward more advanced disease in the NAb^-^ vaccinees, since all patients who had higher Rai stages (III–IV), increased serum β2M levels (>2.4 ml/L), and IVIg requirements failed to develop NAbs (Fig 4D, Table 5). These disease characteristics also correlated with poor D614G NAb responses when the entire CLL cohort was analyzed (Table 3). Among treatment-naïve BNT162b2 vaccinees, elevated serum β2M levels were associated with a 3.5-fold higher risk of failing to mount a D614G NAb response (95% CI 1.8 to 7.2, *p* = 0.003). Limited numbers of treatment-naïve BNT162b2 vaccinees with Rai stages III–IV (*n* = 4) or a requirement for IVIg (*n* = 4), did not provide enough power to test this same association although 4 of 4 CLL patients failed to develop NAbs in both cases. When the presence of a higher Rai stage or IVIg requirement was considered as a proxy for advanced disease, all 6 patients who had either of these traits failed to develop NAbs, suggesting a 2.7-fold higher risk (95% CI 1.4 to 4.7, *p* = 0.02). While this post hoc analysis requires further validation, it should be noted that 7 patients with the same traits were found among the mRNA-1273 vaccinees, yet 5 of these developed a NAb response (S1 and S2 Tables). In addition, male sex emerged as a difference (RR 2.9, 95% CI 1.1 to 8.7, *p* = 0.03), but only when NAb titers to the Delta variant were compared (Fig 4D, Table 5).

**Table 5:** Associations of clinical characteristics with neutralizing responses in treatment-naïve BNT162b2 CLL vaccinees.

	D614G ID_50_			Delta ID_50_		
Characteristic	NAb^+^ (*n* = 15)	NAb^-^ (*n* = 15)	*p*-value	NAb^+^ (*n* = 13)	NAb^-^ (*n* = 17)	*p*-value
Age in years, median (IQR)	70 (65–74)	66 (61–80)	0.8^1^	70 (61–72)	71 (62–81)	0.7^1^
Age ≥65 years, number (%)	11 (73)	10 (67)	1	9 (69)	12 (71)	1
Sex, male, number (%)	5 (33)	9 (60)	0.3	3 (23)	11 (65)	**0.03**
Months from diagnosis to vaccination, median (IQR)	81 (35–100)	62 (32–85)	0.5^1^	62 (34–100)	74 (34–90)	0.9^1^
Days from second vaccination to testing, median (IQR)	54 (31–86)	76 (30–90)	0.9^1^	38 (30–81)	76 (33–90)	0.6^1^
**Rai stage, number (%)**						
III–IV	0/15 (0)	4/15 (27)	0.1	0/13 (0)	4/17 (24)	0.1
**IVIg therapy**						
Number (%)	0/15 (0)	4/15 (27)	0.1	0/13 (0)	4/17 (24)	0.1
**Laboratory parameters, number (%)**						
Absolute lymphocyte count ≥5 × 10^9^/L	11/15 (73)	15/15 (100)	0.1	10/13 (77)	16/17 (94)	0.3
β2-microglobulin >2.4 mg/L	0/15 (0)	6/12 (50)	**0.003**	0/13 (0)	6/14 (43)	**0.02**

^1^ Mann–Whitney test used.

Significant *p*-values are in bold.

CLL, chronic lymphocytic leukemia; ID_50_, reciprocal half-maximal inhibitory dilution; IQR, interquartile range; IVIg, intravenous immunoglobulin; NAb, neutralizing antibody; NS, not significant; Pfizer-BioNTech, BNT162b2.

### Treatment-naïve CLL vaccinees who are unable to mount NAb responses have lower numbers of CD4^+^ T cells

**Fig 5:**
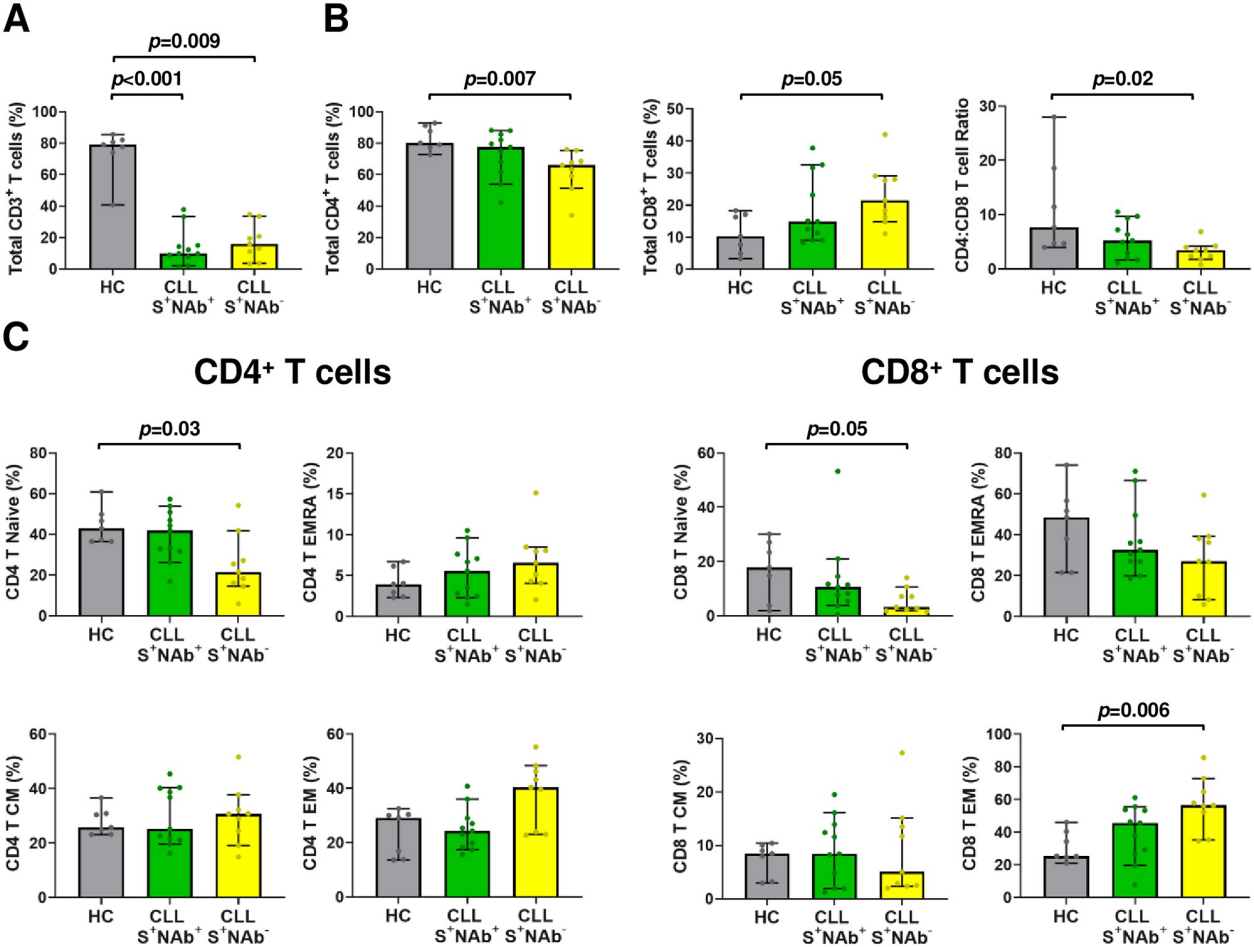
Treatment-naïve CLL vaccinees who are unable to mount NAb responses have reduced naive CD4^+^ T cells and increased effector memory CD8^+^ T cells. (A) Total CD3^+^ as well as (B) CD4^+^, CD8^+^ T cell subsets and CD4:CD8 ratios in SARS-CoV-2 vaccinated HCs ≥65 years old (*n* = 7) and treatment-naive CLL patients who did (S^+^NAb^+^, *n* = 11) or did not (S^+^NAb^-^, *n* = 9) mount a NAb response. (C) CD4^+^ or CD8^+^ naïve, central memory (CM), effector memory (EM), and effector memory CD45RA^+^ (EMRA) T cell subpopulations in SARS-CoV-2 vaccinees as in A and B. Bars indicate the median with 95% CI. Calculations of *p*-values were determined by Dunn’s test of multiple comparisons following a Kruskal–Wallis test. CD45RA, cluster of differentiation 45 including the A protein region; CI, confidence interval; CLL, chronic lymphocytic leukemia; CD4, cluster of differentiation 4; CD8, cluster of differentiation 8; CD3, cluster of differentiation 3; HC, healthy control; NAb, neutralizing antibody; SARS-CoV-2, Severe Acute Respiratory Syndrome Coronavirus 2; S, spike.

Interactions between CD4^+^ T and B cells are critical for germinal center reactions [56,57]. Because these lymphocyte subsets decline as a function of both age and CLL disease [2,58,59], we compared T cell frequencies in age-matched healthy controls (*n* = 7) as well as treatment-naïve SARS-CoV-2 vaccinees who did (S^+^NAb^+^, *n* = 11) versus did not (S^+^NAb^-^, *n* = 9) mount a NAb response. As found for the entire CLL cohort (S2 Fig), we observed a reduction of the total number of CD3^+^ T cells in both S^+^NAb^+^ and S^+^NAb^-^ treatment-naïve CLL patients relative to the healthy controls (*p* < 0.001 and *p* = 0.009; Fig 5A). However, S^+^NAb^-^ vaccinees had significantly lower total CD4^+^ T cell numbers (*p* = 0.007) as well as a trend toward higher CD8^+^ T cell numbers, as reflected by lower CD4:CD8 ratios (*p* = 0.02; Fig 5B). Similar trends were also evident for naïve and memory CD4^+^ and CD8^+^ subsets (Fig 5C). Compared to controls, naïve CD4^+^ T cells were significantly lower in S^+^NAb^-^ (*p* = 0.03), but not S^+^NAb^+^ patients, while there was a coincident rise in effector memory CD8^+^ T cells (*p* = 0.006) in the former group (Fig 5C). These data indicate an association between lower naïve CD4^+^ T cell numbers and the inability of treatment-naive CLL patients to generate NAb responses.

## Discussion

**Fig 6:**
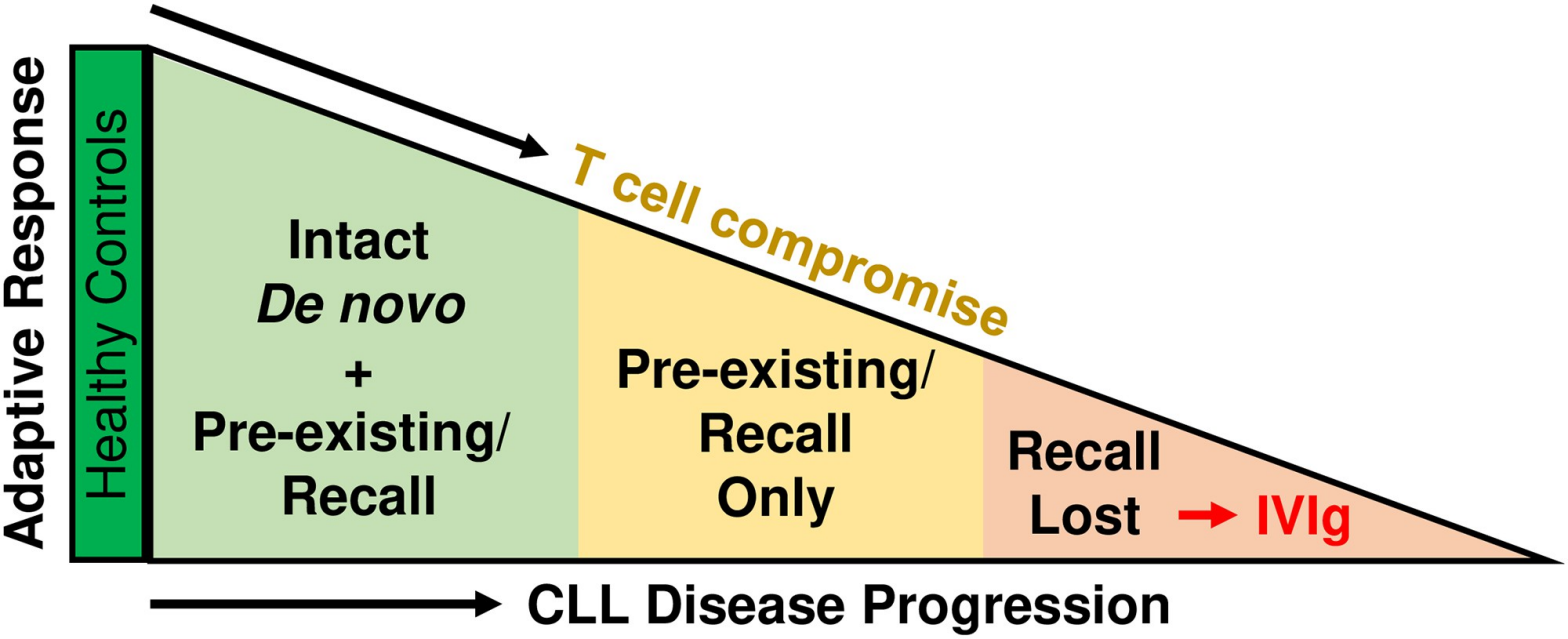
Progressive loss of adaptive immune functions in treatment-naive CLL patients. SARS-CoV-2 vaccination uncovered 3 distinct profiles in CLL patients that reflect a graded decline in adaptive immune function. CLL, chronic lymphocytic leukemia; IVIg, intravenous immunoglobulin; SARS-CoV-2, Severe Acute Respiratory Syndrome Coronavirus 2.

CLL is typically a slowly advancing B cell lymphoproliferative disorder that ultimately impairs the ability of affected individuals to combat infections. Here, we dissected humoral and cellular immune responses to SARS-CoV-2 vaccination in a clinically well-characterized cohort of CLL patients to gain information about the extent and severity of their immune impairment. Comparing the immunogenicity of 2 mRNA (BNT162b2 and mRNA-1273) vaccines, we discovered a previously unappreciated deterioration of adaptive immune functions, which progressed inexorably over the course of CLL disease (Fig 6). By examining both binding and neutralizing antibody responses, we found a subset of mostly treatment-naive vaccinees who were still able to mount de novo responses to SARS-CoV-2 antigens, albeit at titers lower than healthy controls (S^+^NAb^+^, light green). A second group of CLL vaccinees was unable to generate SARS-CoV-2 neutralizing antibodies (S^+^NAb^-^, yellow), but had spike-binding antibodies that primarily reacted with the SARS-CoV-2 S2 subunit. Although these antibodies could have been vaccine-induced, it is much more likely that they represent recall responses of preexisting anti-HCoV antibodies that cross-react with conserved S2 epitopes. The latter possibility is reminiscent of antigenic imprinting [60], which refers to the preferential reactivation of cross-reactive memory B cells from an initial antigenic exposure, rather than the initiation of de novo responses when encountering a new related antigen. The fact that S^+^NAb^-^ vaccinees had more advanced disease, with lower naïve CD4^+^ and higher CD8^+^ effector memory T cells, is consistent with this interpretation. The third group of CLL vaccinees had no detectable SARS-CoV-2 antibodies (S^-^NAb^-^, light red), indicating an inability to mount de novo as well as recall responses. Most of these individuals required IVIg prophylaxis, demonstrating they were the most immune compromised. Thus, SARS-CoV-2 vaccination exposed a progressive loss of immune functions in CLL patients, including those not meeting the criteria for therapy, with preexisting memory being preserved longer than the capacity to respond to new antigens (Fig 6).

Although we cannot exclude that some S^+^NAb^-^ CLL patients mounted vaccine-induced de novo responses, the absence of detectable neutralizing antibodies suggests that these individuals lacked key immune elements required for the induction of germinal center B cell responses and antibody affinity maturation. This disparate humoral response was accompanied by diminished frequencies and altered functions of T cells that were more biased towards a CD8^+^ response. Nonetheless, S^+^NAb^-^ CLL vaccinees maintained HCoV-specific antibodies at levels comparable to healthy controls. Thus, SARS-CoV-2 vaccination in the context of a partially compromised immune system may favor reactivation of preexisting memory over the stimulation of naïve B cells, similar to what has been observed for responses to influenza following vaccination in the elderly [59]. Unfortunately, we were unable to experimentally test this possibility because pre-vaccination samples were not available. Given the essential contributions of naïve B and CD4^+^ T cells to de novo responses, their decline over the CLL disease course is expected to worsen the capacity for engaging neoantigens. Germinal center-based functions would be increasingly diminished and the potential for generating new responses would eventually be lost. Our study thus suggests that vaccination against SARS-CoV-2 and other neoantigens could be used as a tool to assess the status of this decline and to gain greater insight into the specific mechanisms contributing to the immune impairment.

Recent studies have shown that CLL patients benefit from adjuvanted zoster vaccines, including individuals on chronic BTK inhibition, but that hepatitis B immunization elicited poor or no responses in both treatment-naive and BTK inhibitor treated patients [8]. The effectiveness of pneumococcal and influenza immunization is also low in treatment-naive CLL patients, but has been observed to improve with higher dosing, adjuvant-conjugation, and earlier administration following diagnosis [6,7,61]. These results suggest that CLL patients with a reduced ability to mount de novo responses may benefit from more rationally designed vaccine regimens. Indeed, about a quarter of CLL patients who failed to respond to 2 SARS-CoV-2 mRNA immunizations, subsequently seroconverted following a third immunization [62,63]. Thus, CLL patients should be considered candidates for alternative vaccination regimens. For example, the high-dose flu vaccine, which is tailored for the elderly [64], may also elicit stronger and broader responses in CLL patients with partially impaired adaptive immunity. Similarly, a COVID vaccine that includes more than 1 variant may improve de novo responses. Clinical trials that formally test these possibilities should be of high priority.

For treatment-naive patients who lack humoral responses (S^-^NAb^-^) or those on B cell targeted therapies that inhibit seroconversion or NAb generation, passive immunotherapies and antivirals will continue to be important. More than 25% of CLL patients require IVIg [9] and recent studies confirm the presence of SARS-CoV-2 NAbs in US preparations [65]. Prophylactic administration of recombinant neutralizing antibodies has also proven beneficial in the immunocompromised prior to the emergence of variants of concern that escape neutralization [66,67]. Since it is difficult for these treatments to keep up with the pace of viral diversification, a cocktail of prophylactic recombinant neutralizing antibodies specifically engineered for both breadth and durability would be a critical advance for this patient population.

A key finding in our study was the demonstration of superior neutralizing antibody responses in CLL patients who received the mRNA-1273 vaccine. This observation is consistent with previous findings of improved seroconversion rates, binding antibody titers, and T cell activity for mRNA-1273 vaccinees in studies of other hematologic malignancies, including CLL [31,63,68]. Our data may have broader relevance to immunocompromised populations who are estimated to make up 2.7% of the adult population [69]. A meta-analysis of 82 COVID-19 vaccination studies in immunocompromised patients with solid tumors, hematologic malignancies, rheumatic, and auto-inflammatory conditions [70] showed that seroconversion rates as well as the magnitude of binding antibody titers were significantly lower compared to immunocompetent persons. Importantly, lower vaccine effectiveness as measured by breakthrough infections or hospitalization extends to individuals with multiple underlying conditions and comorbidities including the elderly [71–73]. In this context, it is of interest that in other immunocompromised populations the mRNA-1273 vaccine also induced superior humoral responses compared to BNT162b2, including spike and RBD antibody titers and durability [74–78], as well as clinical vaccine effectiveness [76,79,80]. While less is known about neutralizing responses, the superiority of mRNA-1273 found for CLL here appears to extend more broadly to other high-risk populations.

The higher neutralizing response rates and titers in mRNA-1273 vaccinees are likely due, at least in part, to the vaccine dose, which is approximately 3.3-fold higher than that of the BNT162b2 vaccine [81,82]. Indeed, in the elderly, a 100 μg mRNA-1273 dose elicited higher binding and neutralizing antibody titers than a 25 μg dose [83]. However, the 2 vaccines also differ in other properties, including their formulation [84]. Regardless of the reasons, the fact that the mRNA-1273 vaccine elicited higher NAb titers in a larger fraction of CLL patients suggests that this vaccine may confer greater protection from SARS-CoV-2 in this vulnerable population. However, the mRNA-1273 vaccine provided only moderate protection against omicron variants in immunocompromised patients, even after 3 doses [85], with vaccine effectiveness estimated at only 29% [86]. Thus, in addition to vaccine dose and formulation, vaccines will likely need to be updated to target newly emerging escape variants [87,88].

Our observational study has limitations, which will require future studies of larger numbers of participants. Since patients were recruited on a rolling basis, blood samples were not available for pre-vaccination time points. The size of our cohort also did not allow for treatment-specific comparisons (e.g., BTKi or venetoclax) or a more in-depth characterization of treatment-naïve individuals. Given the poor T cell response rates, the study was not adequately powered to define clinical correlates of cellular immunity. Finally, it will be important to determine the impact of SARS-CoV-2 infection on the quantity and quality of SARS-CoV-2 specific immune responses in CLL patients with varying disease progression, which was not possible in the present study.

In summary, our study of SARS-CoV-2 vaccine-induced humoral and cellular responses in CLL patients provides greater granularity of their remaining immune functions, with preexisting immunity being preserved longer than the capacity to mount de novo responses. Most importantly, our finding of the S^+^NAb^-^ serotype in roughly a quarter of CLL vaccinees indicates that “seroconversion” does not necessarily indicate the mounting of a protective antibody response. However, higher NAb titers and response rates identified mRNA-1273 as a potentially superior vaccine for CLL patients. Future studies should consider the utility of vaccination to gauge the extent of disease-induced immune dysfunction in CLL patients and to gain greater insight into the underlying mechanisms.

## Supporting information

S1 STROBE Checklist Checklist of items that should be included in reports of observational studies. (DOCX)

S1 Text Supplemental methods and materials. (PDF)

S1 Fig Serological responses stratified by age for healthy controls and CLL patients. Comparisons of EC_50_ IgG titers for all Spike (*n* = 65) and (B) RBD (*n* = 51) CLL responders, as determined by endpoint titer reactivity, were compared with HC ≥65 years-old (*n* = 13) or <65 years-old (*n* = 17). For neutralizing activity, ID_50_ titers are shown for all responding CLL patients and HC ≥65 years-old as determined in pseudovirus assays against the D614G (HC = 13 vs. CLL = 40) and Delta (HC = 12 vs. CLL = 35) S variants as well as by ACE2/RBD inhibition (HC = 13 vs. CLL = 28). For comparisons with responding HC <65 years-old, sample numbers were: D614G (HC = 16) and Delta (HC = 16) S variants and ACE2/RBD inhibition (HC = 17). Bars indicate the median with 95% CI. Dotted black lines indicate assay sensitivity cutoffs, specifically, EC_50_ values of <100, ID_50_ values of <20 in the neutralization assays, and >90% ACE2 binding in the RBD-inhibition assay. Calculations of *p*-values were determined using the Mann–Whitney test. CLL, chronic lymphocytic leukemia; EC_50_, half-maximal effective concentration; IgG, immunoglobulin G; S, spike; HC, healthy control; RBD, receptor binding domain; Neut ID_50_, half-maximal neutralizing titers; ACE2, angiotensin-converting enzyme-2; CI, confidence interval.
(PDF)

S2 Fig CLL patients have altered total CD4^+^ and CD8^+^ T cell and subpopulation frequencies. PBMCs from vaccinated HC (*n* = 21) and CLL (*n* = 36) participants were immunophenotyped to determine (A) total CD3^+^, CD4^+^ and CD8^+^ T cell frequencies as well as CD4:CD8 ratios. (B and C) Representative flow cytometry plots and quantitative comparisons of CD4^+^ and CD8^+^ subpopulation frequencies defined by the CCR7 and CD45RA surface markers in HC and CLL participants. Bars indicate the median with 95% CI. Calculations of *p*-values were determined using the Mann–Whitney test. CLL, chronic lymphocytic leukemia; CD4, cluster of differentiation 4; CD8, cluster of differentiation 8; PBMCs, peripheral blood mononuclear cells; HC, healthy control; CD3, cluster of differentiation 3; N, naïve; CM, central memory; EM, effector memory; CD45RA, cluster of differentiation 45 including the A protein region; EMRA, effector memory CD45RA; CCR7, C-C chemokine receptor type 7; CI, confidence interval.
(PDF)

S3 Fig Flow cytometry gating strategies for measuring T cell responses from a CLL participant after S peptide pool stimulation. (A) Gating strategy to examine AIM by CD4^+^, cTfh, and CD8^+^ T cells. (B) Gating strategy to examine CD4^+^ and CD8^+^ T cell effector function by ICS. CLL, chronic lymphocytic leukemia; S, spike; CD4, cluster of differentiation 4; cTfh, circulating T follicular helper T cell; CD8, cluster of differentiation 8; ICS, intracellular staining; OX-40, tumor necrosis factor receptor superfamily, member 4; AIM, activation induced marker; PDL1, Programmed death-ligand 1; PD1, Programmed death-1; CXCR5, C-X-C chemokine receptor type 5; CD69, cluster of differentiation 69; CD137, cluster of differentiation 137; CD14, cluster of differentiation 14; CD19, cluster of differentiation 19; CD3, cluster of differentiation 3; IFNγ, interferon gamma; IL2, interleukin 2; TNFα, tumor necrosis factor alpha; CD107a, cluster of differentiation 107a; GrzB, granzyme B; Prf, perforin.
(PDF)

S1 Table Disease characteristics and clinical features of SARS-CoV-2 vaccinated CLL participants. (PDF)

S2 Table Binding and neutralizing antibody titers in the plasma of SARS-CoV-2 vaccinated CLL patients and healthy controls. (PDF)

S3 Table Humoral immune responses for SARS-CoV-2 vaccinated healthy controls and CLL patients by disease/treatment status. (PDF)

S4 Table Associations of serologic responses for SARS-CoV-2 vaccinated healthy controls and CLL patients by disease/treatment status. (PDF)

S5 Table Univariate analysis of serologic responses for SARS-CoV-2 vaccinated CLL patients by disease/treatment status. (PDF)

S6 Table Clinical and disease characteristics of participants included in studies of T cell immunity. (PDF)

S7 Table Subset frequencies, SARS-CoV-2 S peptide-specific AIM responses, and effector functions in healthy control and CLL participant T cells. (PDF)

## Abbreviations

Ab: antibody
ACE2: angiotensin-converting enzyme 2
AIM: activation-induced marker
ALC: absolute lymphocyte count
BTK: Bruton’s tyrosine kinase
CLL: chronic lymphocytic leukemia
COMPASS: combinatorial polyfunctionality analysis of antigen-specific T cell subsets
COVID-19: Coronavirus Disease of 2019
CR: clinical remission
ELISA: enzyme-linked immunosorbent assay
FISH: fluorescence in situ hybridization
ICS: intracellular staining
IVIg: intravenous immunoglobulin
IWCLL: International Workshop on Chronic Lymphocytic Leukemia
MERS-CoV: Middle East Respiratory Syndrome Coronavirus
NAb: neutralizing antibody
PBMC: peripheral blood mononuclear cell
RBD: receptor-binding domain
SARS-CoV-2: Severe Acute Respiratory Syndrome Coronavirus 2
SEB: staphylococcal enterotoxin B
