# Supplementary material for: Exposure of progressive immune dysfunction by SARS-CoV-2 mRNA vaccination in patients with chronic lymphocytic leukemia: A prospective cohort study
Source: PLoS Med. 2023 Jun 29;20(6):e1004157. doi: 10.1371/journal.pmed.1004157 (PMC10309642; doi:10.1371/journal.pmed.1004157)
Supplement: S1 Text — (PDF) [file pmed.1004157.s002.pdf]

S1 Text. Supplemental methods and materials.

SARS-CoV-2 S-protein and subunit ELISA

Plasma IgG binding antibodies to the entire SARS-CoV-2 spike (S) protein as well as receptor binding domain (RBD), and S1 and S2 proteins, were detected by enzyme-linked immunosorbent assay (ELISA) using recombinantly expressed (Wuhan-Hu-1 S-protein and RBD) and purchased (Wuhan-Hu-1 S1 and S2 proteins, ACROBiosystems) proteins as previously described [1-3]. Briefly, Costar high binding flat-bottom 96-well plates were coated with 300 ng per well of a pre-fusion stabilized (S-2P) S protein (residues 1–1138) (plasmid kindly provided by Philip Brouwer and Rogier W. Sanders, Department of Medical Microbiology, University of Amsterdam, Amsterdam, The Netherlands) or 400 ng of recombinantly expressed RBD (residues 419–541), S1 (residues 16-685) or S2 protein (residues 686-1213 with F817P, A892P, A899P, A942P, K986P and V987P substitutions for stabilization) in PBS overnight at 4°C and then incubated with blocking buffer (5% non-fat milk powder in PBS + 0.05% Tween 20) for 1 h at 37°C. Plasma samples were heat-inactivated at 56°C for 1 hour, 5-fold serially diluted in blocking buffer and then added to the plates for 1 h at 37°C. After five washes with PBS-T (PBS + 0.1% Tween 20), plates were incubated for 1 h at 37°C with horseradish peroxidase (HRP)-conjugated goat-anti-human IgG detection antibodies diluted 1:5,000 in blocking buffer. After five additional washes, 3, 3′, 5, 5′-tetramethylbenzidine (TMB) substrate was added for color development for 10 min before the reaction was stopped with an equal volume of 1N H_2SO_4. Absorbance was read at 450 nm using a Synergy 4 spectrophotometer. The average OD_{450} value from three background control wells (no plasma) was subtracted from the protein coated wells. In addition, the average OD_{450} value (plus two standard deviations) of 28 pre-pandemic sera was subtracted from each plasma dilution. Midpoint (EC_{50}) and endpoint titers were determined as described [3,4]. Briefly, midpoint (EC_{50}) titers were calculated by a nonlinear-regression fit of a 4-parameter sigmoid function to the corrected OD_{450} values and the

logarithmic dilution factors (the lower plateau was set to 0; GraphPad Prism software). End-point titers were read from the fitted curve at a corrected OD_{450} cutoff of 0.1.

SARS-CoV-2 pseudovirus neutralization assay

Plasma samples of vaccinees were tested for neutralizing responses against the SARS-CoV-2 variants D614G [5] and B.1.617.2 (also termed Delta with mutations T19R, G142D, ΔE156, ΔF157, R158G, L452R, T478K, D614G, P681R, and D950N compared to Wuhan-Hu-1) [6,7], using an HIV-1 based pseudovirus assay as previously described [8]. Briefly, pseudovirus stocks were generated by co-transfecting spike expression plasmids (encoding proteins with a 19 amino acid cytoplasmic tail deletion) with an HIV-1 nanoluciferase encoding reporter backbone in HEK293T cells. Pseudovirus stocks were tittered to identify the appropriate infectious dose, incubated with five-fold serial dilutions of vaccinee plasma and then used to infect 1.5×10^4 293T clone 13 cells expressing ACE2. Two days post-infection, cells were washed with PBS, lysed, and nanoluciferase activity was determined using a Nano-Glo Luciferase Assay System. Luciferase activity in wells with virus and no plasma were set to 100%, and the dilution of plasma at which luminescence was reduced to 50% (Inhibitory Dose 50; ID_{50}) was calculated as an average of two technical replicates. Each vaccinee plasma was analyzed under anonymized code on at least two occasions, with the geometric mean of all measurements reported. Values below a titer of 1:20 were treated as 20 when averaging.

ACE2/RBD binding inhibition assay

Plasma samples were analyzed with an ACE2/receptor binding domain (RBD) binding inhibition assay as previously described [4,9]. High-binding 96-well plates (Corning #3690) were coated with 50 µl per well of recombinant RBD (Wuhan-Hu-1, RayBiotech) diluted at 1 µg/ml in PBS at

4°C overnight. The following day, plates were washed 3 times with PBS + 0.1% Tween 20 (PBST), and wells were blocked with 100 µl per well of 3% non-fat dry milk in PBST by incubation at room temperature for 1 h. After washing the blocked wells 3 times with PBST, either 50 µl of plasma serially diluted in 1% non-fat dry milk in PBST, or 1% non-fat dry milk in PBST alone as a no inhibition control, was added to wells, and incubated at room temperature for 2 h. Heat-inactivated plasma samples (56°C for 30 min) were initially diluted at 1:25, then serially diluted 2-fold for the assay to 1:400. After incubation, plates were washed 3 times with PBST, then 50 µl of recombinant human ACE2 (RayBiotech) diluted at 0.1 µg/ml in PBST was added to the wells. Plates were incubated at room temperature for 1 h, washed 4 times with PBST, and 50 µl of biotinylated goat anti-human ACE2 (R&D) diluted at 0.1 µg/ml in PBST was added to the wells. Plates were incubated at room temperature for 1 h, washed 4 times with PBST, and then 50 µl of HRP-conjugated streptavidin (Southern Biotech) (1:2,000 in PBST) was added to the wells, and incubated at room temperature for 30 min. Plates were washed 5 times with PBST, developed with 50 µl per well of 3,3′,5,5′-tetramethylbenzidine TMB substrate (Biolegend) at room temperature for 8 min, and the reaction was stopped by addition of 50 µl of 1N H_2SO_4. The OD was measured at 450 nm with a SPECTROstar omega (BMG Labtech) microplate reader. ACE2 binding was expressed as a percentage of OD values relative to the OD_{450} value of a no inhibition control. Binding values of <90% at a 1:25 dilution of plasma were used to calculate inhibitory activity. The upper limit of the assay was 100%.

Human Coronavirus (HCoV) spike protein ELISA

Vaccinee plasma samples were also tested for IgG binding antibodies to spike proteins from endemic HCoVs by ELISA as described [4]. High-binding 96-well plates (Corning #3690) were coated at 4°C overnight with 50 µl per well of the following recombinant Spike (S1+S2) proteins: SARS-CoV, MERS-CoV, HCoV-HKU1, HCoV-OC43, HCoV-NL63, or HCoV-229E (all from Sino

Biological) diluted at 2 µg/ml in PBS. The following day, plates were washed 3 times with 0.1% Tween 20 in PBS (PBST). A blocking solution of 100 µl per well of 3% non-fat dry milk in PBST was added and incubated at room temperature for 1 h before washing plates 3 times with PBST. Plasma samples were heat-inactivated at 56°C for 30 min and initially diluted at 1:20, then serially diluted 5-fold in 1% non-fat dry milk in PBST before adding 50 µl per well, and incubating at room temperature for 2 h. Plates were then washed 4 times with PBST, and 50 µl per well of horseradish peroxidase (HRP) conjugated goat anti-human IgG (Southern Biotech #2045-05) diluted at 1:6,000 in PBST was added to the wells and incubated at room temperature for 1 h to measure Spike protein IgG Ab responses. Plates were washed 5 times with PBST, developed with 50 µl per well of HRP substrate 3, 3', 5, 5' tetramethyl benzidine (TMB, Biolegend) at room temperature for 10 min. The reaction was stopped by addition of 50 µl per well of 1N H_2SO_4. The optical density (OD) was measured at 450 nm with an Epoch microplate spectrophotometer (BioTek). OD_{450} values of blank wells, which were Spike protein-coated wells without plasma, were subtracted from OD_{450} values of sample wells, and EC_{50} values were determined by a nonlinear-regression fit of a 4-parameter sigmoid function with the corrected OD_{450} values and the dilution factors.

Flow cytometry-based T cell immunophenotyping, AIM, and ICS analyses

For immunophenotyping, cryopreserved PBMCs were thawed and washed with FACS wash buffer (2% FBS in PBS). Cells were then stained with CCR7-PerCP5.5, incubated at 37˚C for 20 min, and then stained with the following antibodies: CD3-Alexa780, CD4-BV711, CD8-FITC, CD45RA-BV510, CD19-BUV563, and LIVE/DEAD-UV. After incubation at 4˚C for 30 min, cells were washed twice with FACS wash buffer (2% FBS in PBS) and fixed in a 4% formalin solution. Events were collected on a BD FACSymphony A3 instrument within 24 h and analyzed using FlowJo software (v10).

For activation-induced marker staining (AIM), antigen-specific T cells were measured as previously described [10]. PBMCs were stimulated with SARS-CoV-2 N and S protein peptide pools (BEI Resources) at an individual peptide concentration of 1 μg/ml in the presence of co-stimulatory anti-CD28 and anti-CD49d antibodies (BD Pharmingen). Cell aliquots from each sample were stimulated with equal amounts of dimethyl sulfoxide (DMSO) as a negative control and staphylococcal enterotoxin B (SEB) as a positive control. After incubation at 37°C for 18 h, cells were washed and stained with the following antibodies: CD4-BV711, CD3-Alexa780, CD8-FITC, CD19-BUV563, OX40-PECy7, PDL1-PE, CXCR5-BV421, PD1-BV785, CD137-BV650, CD69-BUV737, and LIVE/DEAD-UV. Cells were then washed and fixed in 4% formalin. Events were collected on a BD FACSymphony A3 instrument and analyzed using FlowJo software (v10).

Intra-cellular staining (ICS) experiments were performed in parallel with the AIM analysis as previously described [10]. CD107a-FITC was added with the co-stimulatory antibody mix. Cells were incubated for a total of 12 h in total. Staining was conducted in three steps: 1) Surface marker staining for 30 min at 4°C with LIVE/DEAD-UV, CD3-Alexa780, CD4-BV711, CD8-V500, CD14-PercpCy5.5, and CD19-BUV563; 2) Permeabilization with CytoFix/CytoPerm solution (BD Biosciences) for 20 min at 4°C; and 3) ICS for 30 min at 4°C with IFNγ-Alexa700, TNFα-PECy7, IL2-APC, GranzymeB-V450, and Perforin-PE. Finally, cells were washed twice and fixed in 4% formalin. Events were collected on a BD FACSymphony A3 instrument and analyzed using FlowJo software (v10). For both AIM and ICS, positive responses were determined by comparison to an unstimulated control with a threshold above at least three times and higher statistical significance by calculation of Chi-square analysis with Yates' correction (*p*-value <0.05). Combinatorial polyfunctionality analysis (COMPASS) of antigen-specific T-cells was calculated as previously described [11].

References

1. Brouwer PJM, Caniels TG, van der Straten K, Snitselaar JL, Aldon Y, Bangaru S, et al. Potent neutralizing antibodies from COVID-19 patients define multiple targets of vulnerability. Science. 2020;369(6504):643-50. Epub 2020/06/17. doi: 10.1126/science.abc5902. PubMed PMID: 32540902; PubMed Central PMCID: PMCPMC7299281.

2. Ketas TJ, Chaturbhuj D, Portillo VMC, Francomano E, Golden E, Chandrasekhar S, et al. Antibody Responses to SARS-CoV-2 mRNA Vaccines Are Detectable in Saliva. Pathog Immun. 2021;6(1):116-34. Epub 2021/06/18. doi: 10.20411/pai.v6i1.441. PubMed PMID: 34136730; PubMed Central PMCID: PMCPMC8201795.

3. Liu W, Russell RM, Bibollet-Ruche F, Skelly AN, Sherrill-Mix S, Freeman DA, et al. Predictors of Nonseroconversion after SARS-CoV-2 Infection. Emerg Infect Dis. 2021;27(9):2454-8. Epub 2021/07/02. doi: 10.3201/eid2709.211042. PubMed PMID: 34193339; PubMed Central PMCID: PMCPMC8386781.

4. Honjo K, Russell RM, Li R, Liu W, Stoltz R, Tabengwa EM, et al. Convalescent plasma-mediated resolution of COVID-19 in a patient with humoral immunodeficiency. Cell Rep Med. 2021;2(1):100164. Epub 2021/02/02. doi: 10.1016/j.xcrm.2020.100164. PubMed PMID: 33521696; PubMed Central PMCID: PMCPMC7817775.

5. Korber B, Fischer WM, Gnanakaran S, Yoon H, Theiler J, Abfalterer W, et al. Tracking Changes in SARS-CoV-2 Spike: Evidence that D614G Increases Infectivity of the COVID-19 Virus. Cell. 2020;182(4):812-27 e19. Epub 2020/07/23. doi: 10.1016/j.cell.2020.06.043. PubMed PMID: 32697968; PubMed Central PMCID: PMCPMC7332439.

6. Liu C, Ginn HM, Dejnirattisai W, Supasa P, Wang B, Tuekprakhon A, et al. Reduced neutralization of SARS-CoV-2 B.1.617 by vaccine and convalescent serum. Cell. 2021;184(16):4220-36 e13. Epub 2021/07/10. doi: 10.1016/j.cell.2021.06.020. PubMed PMID: 34242578; PubMed Central PMCID: PMCPMC8218332.

7. Wang L, Zhou T, Zhang Y, Yang ES, Schramm CA, Shi W, et al. Ultrapotent antibodies against diverse and highly transmissible SARS-CoV-2 variants. Science. 2021;373(6556). Epub 2021/07/03. doi: 10.1126/science.abh1766. PubMed PMID: 34210892; PubMed Central PMCID: PMCPMC9269068.

8. Schmidt F, Weisblum Y, Muecksch F, Hoffmann HH, Michailidis E, Lorenzi JCC, et al. Measuring SARS-CoV-2 neutralizing antibody activity using pseudotyped and chimeric viruses. J Exp Med. 2020;217(11). Epub 2020/07/22. doi: 10.1084/jem.20201181. PubMed PMID: 32692348; PubMed Central PMCID: PMCPMC7372514.

9. Kumar G, Sterrett S, Hall L, Tabengwa E, Honjo K, Larimer M, et al. Comprehensive mapping of SARS-CoV-2 peptide epitopes for development of a highly sensitive serological test for total and neutralizing antibodies. Protein Eng Des Sel. 2022;35:gzab033. Epub 2022/02/18. doi: 10.1093/protein/gzab033. PubMed PMID: 35174857; PubMed Central PMCID: PMCPMC9005051.

10. Boppana S, Qin K, Files JK, Russell RM, Stoltz R, Bibollet-Ruche F, et al. SARS-CoV-2-specific circulating T follicular helper cells correlate with neutralizing antibodies and increase

during early convalescence. PLoS Pathog. 2021;17(7):e1009761. Epub 2021/07/17. doi: 10.1371/journal.ppat.1009761. PubMed PMID: 34270631; PubMed Central PMCID: PMCPMC8318272.

11. Lin L, Finak G, Ushey K, Seshadri C, Hawn TR, Frahm N, et al. COMPASS identifies T-cell subsets correlated with clinical outcomes. Nat Biotechnol. 2015;33(6):610-6. Epub 2015/05/26. doi: 10.1038/nbt.3187. PubMed PMID: 26006008; PubMed Central PMCID: PMCPMC4569006.
