# Supplementary figures and images for: Exposure of progressive immune dysfunction by SARS-CoV-2 mRNA vaccination in patients with chronic lymphocytic leukemia: A prospective cohort study
Source: PLoS Med. 2023 Jun 29;20(6):e1004157. doi: 10.1371/journal.pmed.1004157 (PMC10309642; doi:10.1371/journal.pmed.1004157)

S1 Fig

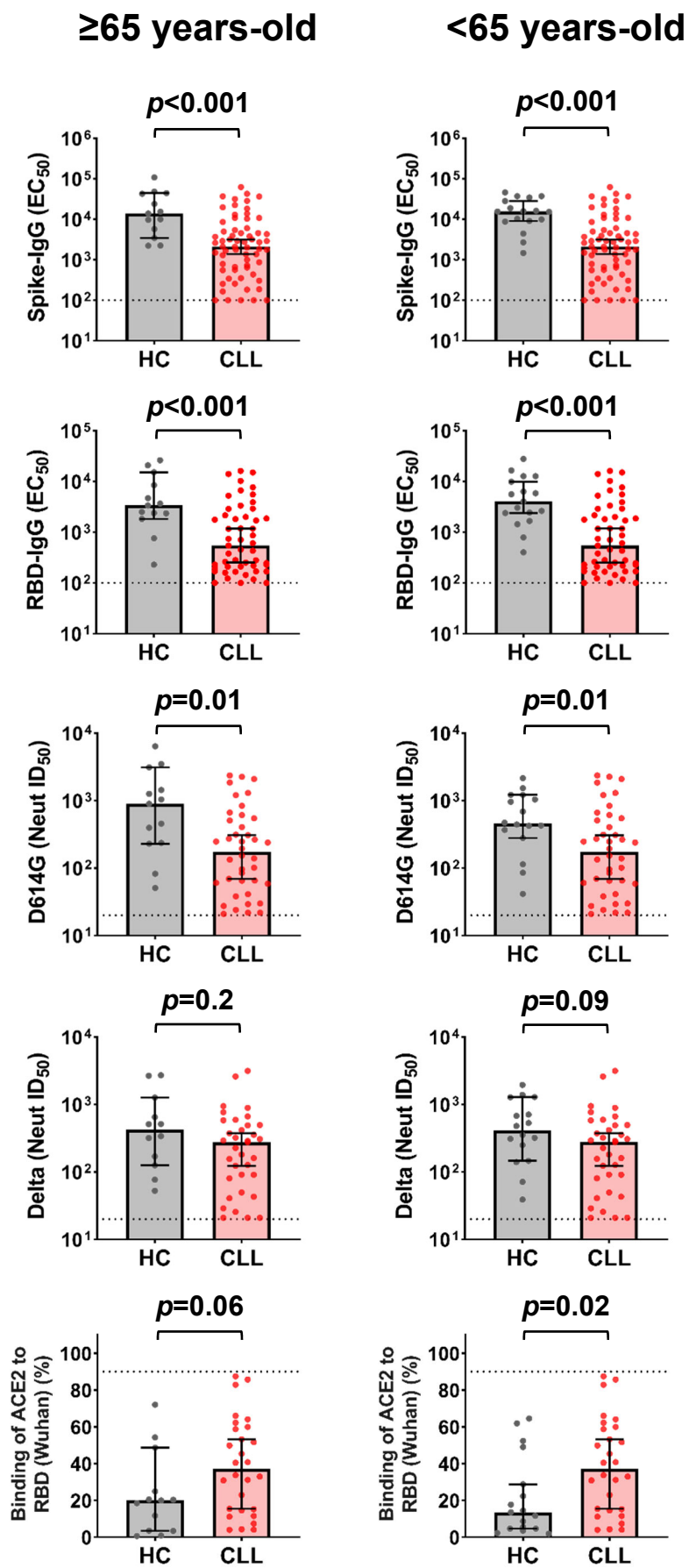

Supplement: S1 Fig — Comparisons of EC50 IgG titers for all Spike (n = 65) and (B) RBD (n = 51) CLL responders, as determined by endpoint titer reactivity, were compared with HC ≥65 years-old (n = 13) or <65 years-old (n = 17). For neutralizing activity, ID50 titers are shown for all responding CLL patients and HC ≥65 years-old as determined in pseudovirus assays against the D614G (HC = 13 vs. CLL = 40) and Delta (HC = 12 vs. CLL = 35) S variants as well as by ACE2/RBD inhibition (HC = 13 vs. CLL = 28). For comparisons with responding HC <65 years-old, sample numbers were: D614G (HC = 16) and Delta (HC = 16) S variants and ACE2/RBD inhibition (HC = 17). Bars indicate the median with 95% CI. Dotted black lines indicate assay sensitivity cutoffs, specifically, EC50 values of <100, ID50 values of <20 in the neutralization assays, and >90% ACE2 binding in the RBD-inhibition assay. Calculations of p-values were determined using the Mann–Whitney test. CLL, chronic lymphocytic leukemia; EC50, half-maximal effective concentration; IgG, immunoglobulin G; S, spike; HC, healthy control; RBD, receptor binding domain; Neut ID50, half-maximal neutralizing titers; ACE2, angiotensin-converting enzyme-2; CI, confidence interval. (PDF) [file pmed.1004157.s003.pdf]

**S2 Fig**

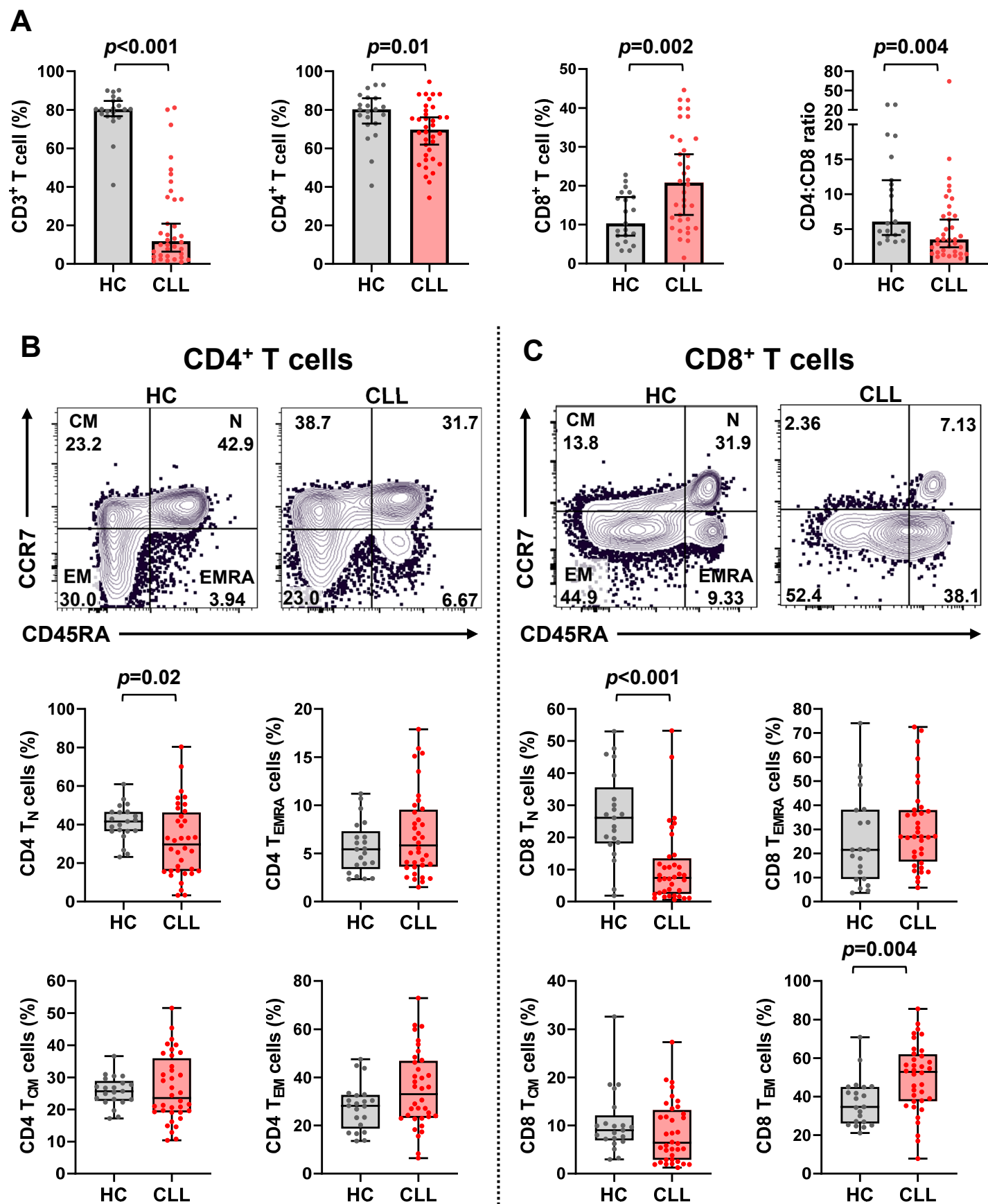

Supplement: S2 Fig — PBMCs from vaccinated HC (n = 21) and CLL (n = 36) participants were immunophenotyped to determine (A) total CD3+, CD4+ and CD8+ T cell frequencies as well as CD4:CD8 ratios. (B and C) Representative flow cytometry plots and quantitative comparisons of CD4+ and CD8+ subpopulation frequencies defined by the CCR7 and CD45RA surface markers in HC and CLL participants. Bars indicate the median with 95% CI. Calculations of p-values were determined using the Mann–Whitney test. CLL, chronic lymphocytic leukemia; CD4, cluster of differentiation 4; CD8, cluster of differentiation 8; PBMCs, peripheral blood mononuclear cells; HC, healthy control; CD3, cluster of differentiation 3; N, naïve; CM, central memory; EM, effector memory; CD45RA, cluster of differentiation 45 including the A protein region; EMRA, effector memory CD45RA; CCR7, C-C chemokine receptor type 7; CI, confidence interval. (PDF) [file pmed.1004157.s004.pdf]

# S3 Fig

**A**

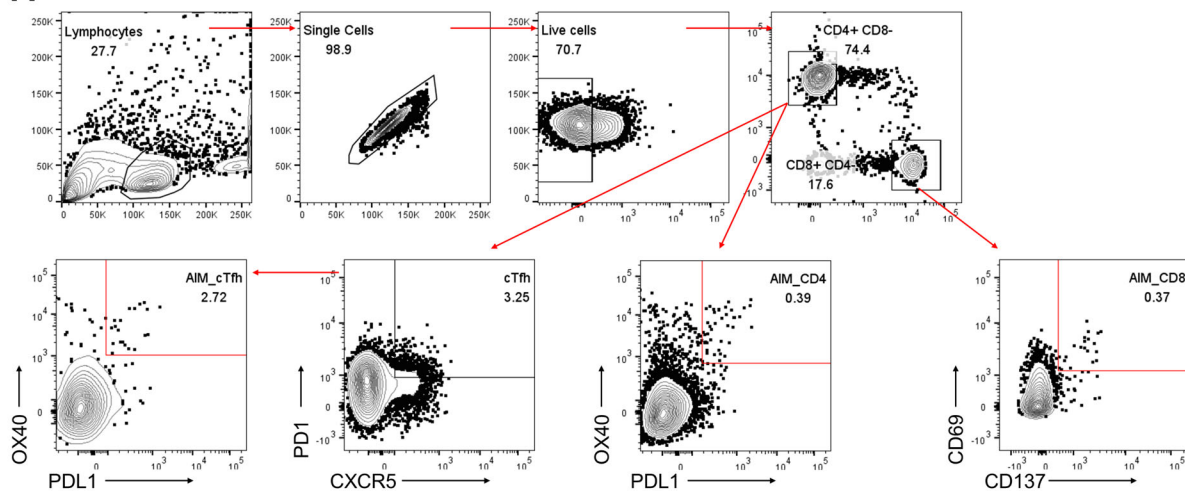

**B**

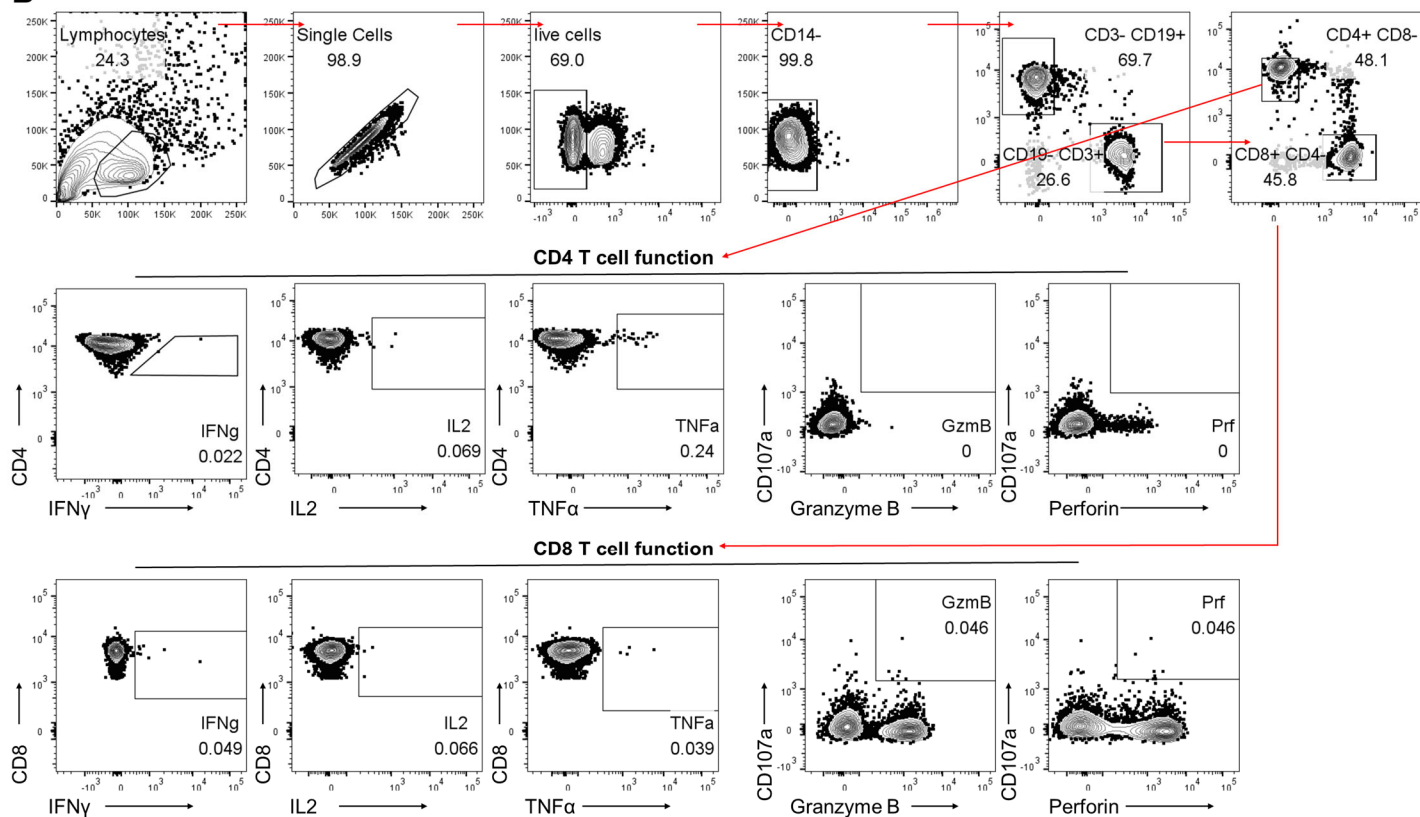

Supplement: S3 Fig — (A) Gating strategy to examine AIM by CD4+, cTfh, and CD8+ T cells. (B) Gating strategy to examine CD4+ and CD8+ T cell effector function by ICS. CLL, chronic lymphocytic leukemia; S, spike; CD4, cluster of differentiation 4; cTfh, circulating T follicular helper T cell; CD8, cluster of differentiation 8; ICS, intracellular staining; OX-40, tumor necrosis factor receptor superfamily, member 4; AIM, activation induced marker; PDL1, Programmed death-ligand 1; PD1, Programmed death-1; CXCR5, C-X-C chemokine receptor type 5; CD69, cluster of differentiation 69; CD137, cluster of differentiation 137; CD14, cluster of differentiation 14; CD19, cluster of differentiation 19; CD3, cluster of differentiation 3; IFNγ, interferon gamma; IL2, interleukin 2; TNFα, tumor necrosis factor alpha; CD107a, cluster of differentiation 107a; GrzB, granzyme B; Prf, perforin. (PDF) [file pmed.1004157.s005.pdf]
