# Supplementary material for: Exposure of progressive immune dysfunction by SARS-CoV-2 mRNA vaccination in patients with chronic lymphocytic leukemia: A prospective cohort study
Source: PLoS Med. 2023 Jun 29;20(6):e1004157. doi: 10.1371/journal.pmed.1004157 (PMC10309642; doi:10.1371/journal.pmed.1004157)
Supplement: S1 Table — (PDF) [file pmed.1004157.s006.pdf]

S1 Table. Disease characteristics and clinical features of SARS-CoV-2 vaccinated CLL subjects.

CLL Sample	Clinical Features			Prognostic Markers and Cytogenetics			Therapeutic History and Type				Humoral Immune Status				Vaccine Features	
	Rai stage[a] 0-I or III-IV	ALC < or ≥5,000 10^6/L	β2M > or ≤2.4 mg/L	*IGHV* MT or UM	CD38 ≥ or <20%	FISH	Any prior therapy	Time from anti-CD20 therapy <12 or ≥12 mo	BTKi alone or combo[b]	Other active therapy	Ig Isotypes: IgM < or ≥40 mg/L	IgG < or ≥650 mg/L	IgA < or ≥60 mg/L	IVIg Tx	Type	Months from second vaccine
**CLLV1**	NA	<5,000	≤2.4	UM	<20%	del(13q)	Yes	<12 mo	Combo	NA	NA	NA	NA	IVIg	Pfizer	≤1
**CLLV2**	Rai 0-I	≥5,000	>2.4	UM	≥20%	tri12	No	NA	NA	NA	<40	<650	≥60	NA	Pfizer	≤1
**CLLV3**	NA	<5,000	>2.4	UM	<20%	del(13q)	Yes	≥12 mo	Alone	NA	<40	<650	≥60	NA	Pfizer	≤1
**CLLV4**	NA	<5,000	>2.4	UM	<20%	del(11q)	Yes	≥12 mo	NA	NA	NA	NA	NA	IVIg	Moderna	≤1
**CLLV5**	NA	<5,000	≤2.4	ND	≥20%	del(17p)	Yes	≥12 mo	Combo	I/BTKi	NA	NA	NA	IVIg	Moderna	≤1
**CLLV6**	NA	≥5,000	>2.4	ND	<20%	del(13q)	Yes	NA	Alone	NA	NA	NA	NA	IVIg	Pfizer	2
**CLLV7**	NA	<5,000	≤2.4	ND	≥20%	tri12	Yes	≥12 mo	Combo	P/BTKi	NA	NA	NA	IVIg	Pfizer	2
**CLLV8**	Rai 0-I	≥5,000	>2.4	MT	<20%	del(13q)	No	NA	NA	NA	NA	NA	NA	IVIg	Moderna	≤1
**CLLV9**	NA	<5,000	ND	MT	<20%	del(13q)	Yes	≥12 mo	NA	NA	NA	NA	NA	IVIg	Pfizer	≤1
**CLLV10**	Rai 0-I	≥5,000	>2.4	ND	≥20%	del(13q)	No	NA	NA	NA	≥40	≥650	≥60	NA	Moderna	≤1
**CLLV11**	NA	<5,000	≤2.4	UM	≥20%	tri12	Yes	<12 mo	NA	NA	<40	≥650	≥60	NA	Pfizer	≤1
**CLLV12**	Rai III-IV	≥5,000	ND	MT	<20%	del(13q)	No	NA	NA	NA	NA	NA	NA	IVIg	Pfizer	≤1
**CLLV13**	NA	≥5,000	>2.4	UM	≥20%	del(11q)	Yes	≥12 mo	Alone	NA	<40	<650	<60	NA	Pfizer	2
**CLLV14**	NA	≥5,000	>2.4	UM	<20%	tri12	Yes	≥12 mo	NA	P	<40	≥650	≥60	NA	Pfizer	≤1
**CLLV15**	Rai 0-I	≥5,000	ND	MT	<20%	del(13q)	No	NA	NA	NA	NA	NA	NA	IVIg	Moderna	≤1
**CLLV16**	NA	<5,000	>2.4	ND	ND	tri12	Yes	<12 mo	Combo	NA	≥40	≥650	≥60	NA	Moderna	≤1
**CLLV17**	NA	<5,000	≤2.4	UM	≥20%	ND	Yes	≥12 mo	Alone	NA	NA	NA	NA	IVIg	Moderna	≤1
**CLLV18**	NA	<5,000	>2.4	MT	<20%	del(13q)	Yes	<12 mo	Combo	A/BTKi/V	<40	≥650	≥60	NA	Pfizer	2
**CLLV19**	Rai 0-I	≥5,000	≤2.4	MT	<20%	ND	No	NA	NA	NA	<40	<650	<60	NA	Moderna	≤1
**CLLV20**	Rai 0-I	≥5,000	>2.4	MT	≥20%	del(13q)	No	NA	NA	NA	<40	<650	≥60	NA	Pfizer	2
**CLLV21**	Rai III-IV	≥5,000	≤2.4	MT	≥20%	WNL	No	NA	NA	NA	<40	<650	≥60	NA	Pfizer	2
**CLLV22**	Rai 0-I	<5,000	>2.4	UM	<20%	del(13q)	Yes	<12 mo	NA	NA	NA	NA	NA	IVIg	Pfizer	2
**CLLV23**	Rai 0-I	≥5,000	>2.4	MT	<20%	WNL	No	NA	NA	NA	≥40	≥650	≥60	NA	Pfizer	2
**CLLV24**	NA	<5,000	>2.4	UM	<20%	WNL	Yes	<12 mo	NA	NA	≥40	≥650	≥60	NA	Moderna	≤1
**CLLV25**	NA	<5,000	≤2.4	ND	<20%	del(13q)	Yes	≥12 mo	NA	C	≥40	<650	<60	NA	Pfizer	2
**CLLV26**	NA	<5,000	>2.4	ND	<20%	tri12	Yes	≥12 mo	Alone	NA	<40	<650	<60	NA	Moderna	≤1
**CLLV27**	Rai 0-I	≥5,000	>2.4	MT	≥20%	del(13q)	No	NA	NA	NA	≥40	≥650	≥60	NA	Pfizer	2
**CLLV28**	Rai 0-I	≥5,000	ND	MT	<20%	WNL	No	NA	NA	NA	<40	<650	≥60	NA	Moderna	2
**CLLV29**	NA	<5,000	≤2.4	UM	<20%	del(13q)	Yes	NA	Alone	NA	<40	<650	≥60	NA	Pfizer	2
**CLLV30**	Rai III-IV	≥5,000	>2.4	MT	<20%	del(17p)	No	NA	NA	NA	<40	≥650	≥60	NA	Moderna	2
**CLLV31**	Rai 0-I	≥5,000	≤2.4	MT	<20%	del(13q)	Yes	NA	NA	NA	NA	NA	NA	IVIg	Pfizer	≤1

**CLLV32**	Rai 0-I	<5,000	>2.4	MT	<20%	del(13q)	No	NA	NA	NA	<40	≥650	≥60	NA	Pfizer	≥3
**CLLV33**	Rai 0-I	<5,000	>2.4	MT	<20%	del(13q)	No	NA	NA	NA	≥40	≥650	≥60	NA	Pfizer	≥3
**CLLV34**	Rai 0-I	≥5,000	≤2.4	MT	<20%	WNL	No	NA	NA	NA	<40	≥650	≥60	NA	Pfizer	≤1
**CLLV35**	NA	<5,000	>2.4	ND	<20%	tri12	Yes	≥12 mo	NA	NA	≥40	≥650	≥60	NA	Pfizer	≤1
**CLLV36**	Rai 0-I	≥5,000	>2.4	MT	<20%	del(13q)	No	NA	NA	NA	NA	NA	NA	IVIg	Pfizer	2
**CLLV37**	NA	<5,000	≤2.4	UM	<20%	del(17p)	Yes	≥12 mo	NA	NA	≥40	≥650	≥60	NA	Pfizer	≤1
**CLLV38**	Rai 0-I	≥5,000	>2.4	MT	<20%	del(17p)	No	NA	NA	NA	≥40	≥650	≥60	NA	Pfizer	2
**CLLV39**	NA	<5,000	>2.4	MT	<20%	del(13q)	Yes	<12 mo	Combo	NA	<40	<650	<60	NA	Pfizer	2
**CLLV40**	Rai III-IV	≥5,000	>2.4	MT	<20%	del(13q)	Yes	≥12 mo	NA	NA	<40	<650	<60	NA	Pfizer	2
**CLLV41**	NA	<5,000	≤2.4	UM	≥20%	del(17p)	Yes	<12 mo	NA	NA	NA	NA	NA	IVIg	Moderna	≤1
**CLLV42**	NA	≥5,000	>2.4	UM	<20%	del(11q)	Yes	≥12 mo	Alone	NA	NA	NA	NA	IVIg	Pfizer	2
**CLLV43**	NA	<5,000	>2.4	ND	<20%	del(13q)	Yes	NA	Alone	NA	≥40	≥650	≥60	NA	Pfizer	2
**CLLV44**	Rai 0-I	<5,000	>2.4	ND	ND	ND	No	NA	NA	NA	<40	≥650	≥60	NA	Pfizer	2
**CLLV45**	NA	<5,000	≤2.4	UM	<20%	del(13q)	Yes	<12 mo	NA	A/PI3Ki/V	<40	<650	≥60	NA	Moderna	2
**CLLV46**	Rai 0-I	≥5,000	>2.4	MT	<20%	del(13q)	No	NA	NA	NA	<40	<650	<60	NA	Pfizer	≤1
**CLLV47**	NA	<5,000	>2.4	ND	ND	tri12	Yes	≥12 mo	NA	NA	≥40	≥650	≥60	NA	Pfizer	2
**CLLV48**	Rai 0-I	≥5,000	≤2.4	MT	<20%	del(13q)	No	NA	NA	NA	≥40	≥650	≥60	NA	Moderna	≥3
**CLLV49**	Rai 0-I	≥5,000	>2.4	UM	≥20%	del(11q)	Yes	≥12 mo	NA	NA	≥40	<650	≥60	NA	Moderna	≤1
**CLLV50**	NA	≥5,000	ND	MT	<20%	del(13q)	Yes	<12 mo	NA	NA	NA	NA	NA	IVIg	Pfizer	2
**CLLV51**	NA	<5,000	≤2.4	UM	≥20%	del(13q)	Yes	<12 mo	NA	NA	<40	<650	<60	NA	Moderna	≤1
**CLLV52**	Rai 0-I	≥5,000	>2.4	MT	<20%	del(13q)	No	NA	NA	NA	<40	≥650	≥60	NA	Pfizer	≥3
**CLLV53**	NA	<5,000	>2.4	ND	≥20%	ND	Yes	≥12 mo	NA	NA	NA	NA	NA	IVIg	Pfizer	≥3
**CLLV54**	Rai 0-I	≥5,000	≤2.4	MT	<20%	del(13q)	Yes	≥12 mo	NA	NA	NA	NA	NA	IVIg	Moderna	2
**CLLV55**	Rai III-IV	≥5,000	ND	UM	≥20%	WNL	No	NA	NA	NA	<40	<650	<60	NA	Pfizer	≤1
**CLLV56**	Rai 0-I	<5,000	>2.4	ND	<20%	del(13q)	No	NA	NA	NA	≥40	≥650	≥60	NA	Moderna	≥3
**CLLV57**	Rai 0-I	≥5,000	>2.4	MT	<20%	del(13q)	No	NA	NA	NA	<40	<650	≥60	NA	Pfizer	2
**CLLV58**	Rai 0-I	≥5,000	>2.4	MT	<20%	WNL	No	NA	NA	NA	<40	≥650	<60	NA	Pfizer	≤1
**CLLV59**	Rai 0-I	≥5,000	ND	MT	<20%	del(17p)	Yes	NA	NA	NA	<40	<650	≥60	NA	Pfizer	≤1
**CLLV60**	Rai III-IV	≥5,000	≤2.4	MT	<20%	tri12	No	NA	NA	NA	NA	NA	NA	IVIg	Pfizer	≥3
**CLLV61**	NA	<5,000	≤2.4	ND	<20%	del(11q)	Yes	NA	Alone	NA	≥40	≥650	≥60	NA	Pfizer	≥3
**CLLV62**	Rai 0-I	≥5,000	>2.4	MT	<20%	WNL	No	NA	NA	NA	<40	≥650	≥60	NA	Moderna	≥3
**CLLV63**	Rai 0-I	<5,000	≤2.4	ND	≥20%	ND	No	NA	NA	NA	<40	≥650	<60	NA	Moderna	≥3
**CLLV64**	NA	<5,000	>2.4	UM	≥20%	del(13q)	Yes	<12 mo	Combo	NA	≥40	≥650	≥60	NA	Pfizer	≤1
**CLLV65**	Rai 0-I	≥5,000	>2.4	MT	<20%	del(13q)	No	NA	NA	NA	<40	<650	≥60	NA	Pfizer	≥3
**CLLV66**	NA	<5,000	≤2.4	UM	≥20%	tri12	Yes	<12 mo	Combo	NA	NA	NA	NA	IVIg	Moderna	≥3
**CLLV67**	NA	<5,000	ND	MT	<20%	del(13q)	Yes	≥12 mo	NA	NA	≥40	≥650	<60	NA	Pfizer	≥3
**CLLV68**	Rai 0-I	≥5,000	≤2.4	UM	<20%	del(11q)	No	NA	NA	NA	<40	≥650	≥60	NA	Pfizer	≥3
**CLLV69**	Rai 0-I	≥5,000	>2.4	MT	<20%	del(13q)	No	NA	NA	NA	<40	≥650	<60	NA	Moderna	≥3

**CLLV70**	NA	≥5,000	≤2.4	MT	<20%	del(13q)	Yes	NA	NA	P	<40	≥650	≥60	NA	Pfizer	≥3
**CLLV71**	NA	<5,000	>2.4	UM	<20%	del(11q)	Yes	≥12 mo	NA	V	NA	NA	NA	IVIg	Moderna	≥3
**CLLV72**	NA	<5,000	>2.4	ND	<20%	WNL	Yes	≥12 mo	NA	NA	≥40	≥650	≥60	NA	Moderna	≥3
**CLLV73**	NA	<5,000	≤2.4	UM	<20%	tri12	Yes	≥12 mo	Alone	NA	NA	NA	NA	IVIg	Pfizer	≥3
**CLLV74**	NA	<5,000	ND	MT	<20%	del(17p)	Yes	NA	Combo	BTKi/V	NA	NA	NA	IVIg	Moderna	≥3
**CLLV75**	NA	≥5,000	ND	MT	<20%	del(13q)	Yes	<12 mo	NA	NA	<40	<650	<60	NA	Moderna	≥3
**CLLV76**	Rai 0-I	≥5,000	>2.4	MT	<20%	del(13q)	No	NA	NA	NA	≥40	≥650	≥60	NA	Moderna	≥3
**CLLV77**	NA	<5,000	≤2.4	UM	≥20%	del(11q)	Yes	≥12 mo	NA	L	NA	NA	NA	IVIg	Moderna	≥3
**CLLV78**	Rai 0-I	≥5,000	≤2.4	MT	ND	del(13q)	No	NA	NA	NA	<40	≥650	≥60	NA	Pfizer	≥3
**CLLV79**	Rai 0-I	≥5,000	ND	MT	<20%	del(13q)	No	NA	NA	NA	<40	≥650	≥60	NA	Pfizer	≥3
**CLLV80**	Rai 0-I	≥5,000	≤2.4	UM	<20%	del(13q)	Yes	≥12 mo	NA	NA	<40	<650	≥60	NA	Moderna	2
**CLLV81**	NA	<5,000	≤2.4	UM	<20%	del(11q)	Yes	≥12 mo	Alone	NA	<40	<650	≥60	NA	Pfizer	≥3
**CLLV82**	Rai 0-I	≥5,000	>2.4	MT	<20%	del(13q)	No	NA	NA	NA	<40	≥650	≥60	NA	Moderna	≥3
**CLLV83**	NA	<5,000	ND	ND	<20%	WNL	Yes	<12 mo	Combo	NA	<40	<650	≥60	NA	Moderna	2
**CLLV84**	Rai 0-I	≥5,000	>2.4	MT	≥20%	del(13q)	No	NA	NA	NA	≥40	≥650	≥60	NA	Pfizer	2
**CLLV85**	Rai 0-I	≥5,000	>2.4	MT	ND	del(13q)	No	NA	NA	NA	<40	≥650	≥60	NA	Pfizer	2
**CLLV86**	Rai 0-I	≥5,000	>2.4	MT	≥20%	tri12	No	NA	NA	NA	≥40	<650	≥60	NA	Pfizer	≥3
**CLLV87**	Rai 0-I	≥5,000	≤2.4	MT	<20%	tri12	No	NA	NA	NA	NA	NA	NA	IVIg	Pfizer	≥3
**CLLV88**	Rai 0-I	≥5,000	>2.4	UM	≥20%	del(11q)	No	NA	NA	NA	≥40	≥650	≥60	NA	Pfizer	2
**CLLV89**	NA	<5,000	ND	ND	<20%	del(13q)	Yes	≥12 mo	NA	NA	≥40	≥650	≥60	NA	Pfizer	≥3
**CLLV90**	Rai 0-I	≥5,000	>2.4	MT	<20%	del(13q)	No	NA	NA	NA	≥40	<650	≥60	NA	Pfizer	≥3
**CLLV91**	Rai 0-I	<5,000	>2.4	ND	<20%	tri12	No	NA	NA	NA	<40	≥650	≥60	NA	Pfizer	≥3
**CLLV92**	NA	<5,000	≤2.4	UM	<20%	tri12	Yes	NA	Alone	NA	<40	<650	≥60	NA	Pfizer	≥3
**CLLV93**	Rai 0-I	≥5,000	>2.4	MT	<20%	del(13q)	No	NA	NA	NA	<40	≥650	≥60	NA	Moderna	≥3
**CLLV94**	Rai 0-I	≥5,000	≤2.4	MT	≥20%	del(13q)	No	NA	NA	NA	<40	≥650	≥60	NA	Moderna	≥3
**CLLV95**	Rai 0-I	≥5,000	>2.4	ND	<20%	del(13q)	No	NA	NA	NA	<40	<650	≥60	NA	Pfizer	≥3

[a]Determined for patients who are treatment-naïve (shaded) or off-treatment in relapse.

[b]Combo indicates concomitant treatment with anti-CD20/BTKi unless otherwise specified in the other active therapy column.

SARS-CoV-2, Severe Acute Respiratory Syndrome Coronavirus-2; CLL, chronic lymphocytic leukemia; NA, not applicable; ALC, absolute lymphocyte count; β2M, beta-2 microglobulin; ND, no data; *IGHV*, immunoglobulin heavy chain variable region gene; MT, mutated; UM, unmutated; CD38, cluster of differentiation 38; FISH, fluorescence *in situ* hybridization; del, deletion; WNL, within normal limits; CD20, cluster of differentiation 20; mo, months: BTKi, Bruton's tyrosine kinase inhibitor; I, imatinib; P, prednisone; A, anti-CD20; V, venetoclax; C, cyclosporine; PI3Ki, Phosphoinositide 3-kinase inhibitor; L, lenalidomide; Ig, immunoglobulin; IgM, immunoglobulin M; IgG, immunoglobulin G; IgA, immunoglobulin A; IVIg, intravenous immunoglobulin; Tx, treatment; Pfizer-BioNTech, BNT162b2; Moderna, mRNA-1273.
