# Supplementary material for: Exposure of progressive immune dysfunction by SARS-CoV-2 mRNA vaccination in patients with chronic lymphocytic leukemia: A prospective cohort study
Source: PLoS Med. 2023 Jun 29;20(6):e1004157. doi: 10.1371/journal.pmed.1004157 (PMC10309642; doi:10.1371/journal.pmed.1004157)
Supplement: S2 Table — (PDF) [file pmed.1004157.s007.pdf]

**S2 Table. Binding and neutralizing antibody titers in the plasma of SARS-CoV-2 vaccinated CLL patients and healthy controls.**

| ID | Status | Gender | Binding antibodies | | | | | | | | RBD/ACE2 binding | Neutralization | |
|---|---|---|---|---|---|---|---|---|---|---|---|---|---|
| | | | Spike | | RBD | | S1 | | S2 | | | D614G | Delta |
| | | | $EC_{50}$ | Endpoint | $EC_{50}$ | Endpoint | $EC_{50}$ | Endpoint | $EC_{50}$ | Endpoint | | $ID_{50}$ | $ID_{50}$ |
| **HC1** | Healthy | Male | 3454 | 66220 | 1829 | 14885 | 2754 | 46347 | 1492 | 13180 | 54.2 | 237 | 171 |
| **HC2** | Healthy | Female | 44369 | >312500 | 15247 | 239875 | 24253 | >312500 | 10506 | 100315 | 0.9 | 3114 | 651 |
| **HC3** | Healthy | Female | 48276 | >312500 | 20806 | >312500 | 38100 | >312500 | 14550 | 207896 | 11.7 | 3493 | 2711 |
| **HC4** | Healthy | Male | 9754 | 94317 | 2396 | 23760 | 3473 | 64473 | 4366 | 74455 | 20.5 | 230 | 125 |
| **HC5** | Healthy | Male | 5780 | 76936 | 2351 | 25047 | 4339 | 61521 | 2355 | 22565 | 19.9 | 897 | 319 |
| **HC6** | Healthy | Female | 9103 | 132297 | 2390 | 25922 | 4722 | 84416 | 3407 | 61522 | 17.6 | 1220 | 1959 |
| **HC7** | Healthy | Female | 23979 | >312500 | 8471 | 103877 | 12452 | 135787 | 4468 | 69473 | 3.5 | 1263 | 1272 |
| **HC8** | Healthy | Male | 2247 | 26478 | 230 | 11830 | 1119 | 15479 | 448 | 14123 | 48.6 | 83 | <20 |
| **HC9** | Healthy | Female | 108316 | >312500 | 26151 | >312500 | 54808 | >312500 | 6334 | 132888 | 0.6 | 6398 | 2661 |
| **HC10** | Healthy | Female | 10050 | 151027 | 2527 | 31974 | 4976 | 110597 | 448 | 13278 | 20.3 | 398 | 77 |
| **HC11** | Healthy | Male | 13974 | 177209 | 3377 | 53091 | 4510 | 82832 | 3048 | 54050 | 18.5 | 1046 | 339 |
| **HC12** | Healthy | Female | 15911 | 250229 | 2439 | 26567 | 5186 | 61195 | 2018 | 19229 | 28.7 | 423 | 147 |
| **HC13** | Healthy | Male | 15433 | 266151 | 4678 | 112835 | 10706 | 130281 | 4662 | 76938 | 24.9 | 1437 | 511 |
| **HC14** | Healthy | Female | 16199 | 262987 | 4069 | 86869 | 7589 | 107134 | 2802 | 46163 | 5.5 | 2156 | 1390 |
| **HC15** | Healthy | Female | 34574 | >312500 | 12719 | 201615 | ND | ND | ND | ND | 1.9 | 471 | 479 |
| **HC16** | Healthy | Female | 37503 | >312500 | 16553 | 184510 | ND | ND | ND | ND | 4.6 | 1045 | 1288 |
| **HC17** | Healthy | Female | 18933 | >312500 | 5864 | 91555 | ND | ND | ND | ND | 2.2 | 427 | 353 |
| **HC18** | Healthy | Male | 15276 | 225339 | 6343 | 87278 | ND | ND | ND | ND | 8.7 | 691 | 533 |
| **HC20** | Healthy | Male | 28325 | >312500 | 9890 | 120823 | ND | ND | ND | ND | 3.5 | 964 | 709 |
| **HC21** | Healthy | Male | 37411 | >312500 | 12777 | 160705 | ND | ND | ND | ND | 4.6 | 1221 | 677 |
| **HC22** | Healthy | Male | 9945 | 80034 | 3040 | 44720 | ND | ND | ND | ND | 14.4 | 442 | 320 |
| **HC23** | Healthy | Female | 8790 | 127507 | 2648 | 34505 | ND | ND | ND | ND | 22.3 | 280 | 251 |
| **HC24** | Healthy | Male | 46399 | >312500 | 27919 | >312500 | ND | ND | ND | ND | 11.6 | 1528 | 1295 |
| **HC25** | Healthy | Male | 15586 | 225216 | 5616 | 89684 | ND | ND | ND | ND | 13.5 | 368 | 310 |
| **HC26** | Healthy | Male | 2712 | 45027 | 791 | 15080 | ND | ND | ND | ND | 61.8 | 42 | 39 |
| **HC27** | Healthy | Female | 10379 | 94070 | 1439 | 17079 | ND | ND | ND | ND | 52.3 | 114 | 71 |
| **HC28** | Healthy | Male | 42858 | >312500 | 3682 | 73382 | 8005 | 128500 | 11187 | 116170 | 3.4 | 457 | 514 |
| **HC29** | Healthy | Female | 2230 | 27529 | 763 | 13108 | ND | ND | ND | ND | 72 | 51 | 53 |
| **HC30** | Healthy | Male | 4425 | 96677 | 1669 | 13452 | ND | ND | ND | ND | 49 | 85 | 140 |
| **HC31** | Healthy | Male | 1480 | 15125 | 402 | 7702 | ND | ND | ND | ND | 64.5 | <20 | <20 |

| | | | | | | | | | | | | | |
|---|---|---|---|---|---|---|---|---|---|---|---|---|---|
| **CLLV1** | On Tx | Male | <100 | <100 | <100 | <100 | ND | ND | ND | ND | >90 | <20 | <20 |
| **CLLV2** | Tx naïve | Male | 10814 | 116098 | 2886 | 38114 | ND | ND | ND | ND | 49.8 | 308 | 595 |
| **CLLV3** | On Tx | Female | 438 | 13445 | 159 | 559 | 267 | 2115 | 228 | 6362 | >90 | <20 | <20 |
| **CLLV4** | Off Tx CR | Female | 2097 | 19504 | 433 | 10349 | ND | ND | ND | ND | 59.9 | 59 | 91 |
| **CLLV5** | On Tx | Female | <100 | 3461 | <100 | <100 | <100 | 154 | 179 | 5001 | >90 | <20 | <20 |
| **CLLV6** | On Tx | Female | <100 | <100 | <100 | <100 | ND | ND | ND | ND | >90 | <20 | <20 |
| **CLLV7** | On Tx | Female | <100 | <100 | <100 | <100 | ND | ND | ND | ND | >90 | <20 | <20 |
| **CLLV8** | Tx naïve | Female | 37157 | >312500 | 10300 | 142185 | ND | ND | ND | ND | 11.5 | 2371 | 946 |
| **CLLV9** | Off Tx CR | Female | 870 | 16255 | 247 | 5147 | ND | ND | ND | ND | 82.8 | 38 | 41 |
| **CLLV10** | Tx naïve | Male | 18335 | 262114 | 5265 | 75564 | ND | ND | ND | ND | 11.1 | 840 | 419 |
| **CLLV11** | On Tx | Male | <100 | <100 | <100 | <100 | ND | ND | ND | ND | >90 | <20 | <20 |
| **CLLV12** | Tx naïve | Male | <100 | 533 | <100 | 323 | ND | ND | ND | ND | >90 | <20 | <20 |
| **CLLV13** | On Tx | Female | <100 | <100 | <100 | <100 | ND | ND | ND | ND | >90 | <20 | <20 |
| **CLLV14** | On Tx | Male | <100 | <100 | <100 | <100 | ND | ND | ND | ND | >90 | <20 | <20 |
| **CLLV15** | Tx naïve | Female | <100 | <100 | <100 | <100 | ND | ND | ND | ND | >90 | <20 | <20 |
| **CLLV16** | On Tx | Male | 352 | 6938 | <100 | <100 | 200 | 2552 | 395 | 11303 | >90 | <20 | <20 |
| **CLLV17** | On Tx | Female | <100 | <100 | <100 | <100 | ND | ND | ND | ND | >90 | <20 | <20 |
| **CLLV18** | On Tx | Female | <100 | <100 | <100 | <100 | ND | ND | ND | ND | >90 | <20 | <20 |
| **CLLV19** | Tx naïve | Female | 42825 | >312500 | 15085 | 201882 | ND | ND | ND | ND | 4.1 | 1857 | 490 |
| **CLLV20** | Tx naïve | Male | 184 | 6770 | <100 | <100 | 143 | 4032 | <100 | <100 | >90 | <20 | <20 |
| **CLLV21** | Tx naïve | Male | 13018 | 164471 | <100 | <100 | <100 | <100 | 11869 | 161466 | >90 | <20 | <20 |
| **CLLV22** | Off Tx and R/R | Male | 2022 | 13899 | <100 | <100 | <100 | <100 | 6829 | 110325 | >90 | <20 | <20 |
| **CLLV23** | Tx naïve | Female | 3170 | 37209 | 608 | 12919 | ND | ND | ND | ND | 85.7 | 29 | 26 |
| **CLLV24** | On Tx | Male | <100 | <100 | <100 | <100 | ND | ND | ND | ND | >90 | <20 | <20 |
| **CLLV25** | On Tx | Female | <100 | <100 | <100 | <100 | ND | ND | ND | ND | >90 | <20 | <20 |
| **CLLV26** | On Tx | Male | 10869 | 148175 | <100 | <100 | 1986 | 12518 | 23339 | >312500 | >90 | <20 | <20 |
| **CLLV27** | Tx naïve | Female | 166 | 6293 | <100 | <100 | ND | ND | ND | ND | 53.2 | 314 | 896 |
| **CLLV28** | Tx naïve | Male | 2942 | 51890 | 383 | 10082 | ND | ND | ND | ND | 62.2 | 153 | 276 |
| **CLLV29** | On Tx | Male | <100 | <100 | <100 | <100 | ND | ND | ND | ND | >90 | <20 | <20 |
| **CLLV30** | Tx naïve | Male | 2913 | 43861 | 1809 | 11362 | ND | ND | ND | ND | 65.9 | 265 | 91 |
| **CLLV31** | Off Tx and R/R | Female | <100 | <100 | <100 | <100 | ND | ND | ND | ND | >90 | <20 | <20 |
| **CLLV32** | Tx naïve | Female | 731 | 15965 | 123 | 1405 | ND | ND | ND | ND | >90 | 22 | 21 |
| **CLLV33** | Tx naïve | Male | 254 | 11569 | 253 | 1260 | ND | ND | ND | ND | >90 | 21 | <20 |
| **CLLV34** | Tx naïve | Female | 790 | 15389 | 169 | 1206 | 142 | 5117 | 864 | 19779 | >90 | <20 | <20 |

| | | | | | | | | | | | | | |
|---|---|---|---|---|---|---|---|---|---|---|---|---|---|
| **CLLV35** | Off Tx CR | Female | 342 | 9254 | 171 | 2378 | ND | ND | ND | ND | >90 | 22 | <20 |
| **CLLV36** | Tx naïve | Female | 647 | 13147 | 198 | 4074 | ND | ND | ND | ND | >90 | <20 | <20 |
| **CLLV37** | Off Tx CR | Male | 31919 | >312500 | 14039 | 188186 | ND | ND | ND | ND | 4 | 2095 | 3145 |
| **CLLV38** | Tx naïve | Male | 5424 | 89373 | 1770 | 20417 | ND | ND | ND | ND | 40.5 | 273 | 665 |
| **CLLV39** | On Tx | Male | <100 | <100 | <100 | <100 | ND | ND | ND | ND | >90 | <20 | <20 |
| **CLLV40** | Off Tx and R/R | Female | <100 | <100 | <100 | <100 | ND | ND | ND | ND | >90 | <20 | <20 |
| **CLLV41** | On Tx | Male | <100 | <100 | <100 | <100 | ND | ND | ND | ND | >90 | <20 | <20 |
| **CLLV42** | On Tx | Female | <100 | <100 | <100 | <100 | ND | ND | ND | ND | >90 | <20 | <20 |
| **CLLV43** | On Tx | Female | 1302 | 21707 | <100 | <100 | <100 | 1274 | 3491 | 58888 | >90 | <20 | NA |
| **CLLV44** | Tx naïve | Female | 3175 | 46365 | 549 | 12788 | ND | ND | ND | ND | >90 | 247 | 43 |
| **CLLV45** | On Tx | Female | <100 | <100 | <100 | <100 | ND | ND | ND | ND | >90 | <20 | NA |
| **CLLV46** | Tx naïve | Male | 662 | 12679 | <100 | <100 | <100 | 1784 | 3018 | 45157 | >90 | <20 | <20 |
| **CLLV47** | Off Tx CR | Female | 19885 | 303589 | 3338 | 60781 | ND | ND | ND | ND | 22.9 | 508 | 355 |
| **CLLV48** | Tx naïve | Female | 258 | 4612 | <100 | 1134 | ND | ND | ND | ND | >90 | <20 | <20 |
| **CLLV49** | Off Tx and R/R | Male | 4335 | 80421 | 144 | 4204 | ND | ND | ND | ND | >90 | 69 | 308 |
| **CLLV50** | On Tx | Female | <100 | <100 | <100 | <100 | ND | ND | ND | ND | >90 | <20 | <20 |
| **CLLV51** | On Tx | Male | <100 | <100 | <100 | <100 | ND | ND | ND | ND | >90 | <20 | <20 |
| **CLLV52** | Tx naïve | Female | 3800 | 66371 | 1706 | 14991 | ND | ND | ND | ND | 45.3 | 85 | 50 |
| **CLLV53** | Off Tx CR | Male | 2740 | 29793 | 708 | 16666 | ND | ND | ND | ND | 58.7 | 65 | 156 |
| **CLLV54** | Off Tx and R/R | Female | <100 | <100 | <100 | <100 | ND | ND | ND | ND | >90 | <20 | <20 |
| **CLLV55** | Tx naïve | Male | <100 | 1672 | <100 | <100 | <100 | <100 | <100 | 1841 | >90 | <20 | <20 |
| **CLLV56** | Tx naïve | Male | 2616 | 51938 | 1172 | 19650 | ND | ND | ND | ND | 31.1 | 240 | 293 |
| **CLLV57** | Tx naïve | Female | 6062 | 141781 | 1185 | 16644 | ND | ND | ND | ND | 40.8 | 407 | 123 |
| **CLLV58** | Tx naïve | Female | 4564 | 76566 | 228 | 7036 | ND | ND | ND | ND | >90 | 24 | 21 |
| **CLLV59** | Off Tx and R/R | Male | 305 | 11023 | 166 | 3255 | ND | ND | ND | ND | 64 | 667 | 127 |
| **CLLV60** | Tx naïve | Male | <100 | 100 | <100 | <100 | <100 | <100 | <100 | 137 | >90 | <20 | <20 |
| **CLLV61** | On Tx | Male | <100 | 4297 | <100 | <100 | <100 | <100 | 360 | 13105 | >90 | <20 | <20 |
| **CLLV62** | Tx naïve | Male | 13804 | 179944 | 3920 | 67311 | ND | ND | ND | ND | 14.6 | 1205 | 585 |
| **CLLV63** | Tx naïve | Male | 28720 | >312500 | 6708 | 89175 | ND | ND | ND | ND | 15.2 | 1297 | 227 |
| **CLLV64** | On Tx | Male | <100 | <100 | <100 | <100 | ND | ND | ND | ND | >90 | <20 | <20 |
| **CLLV65** | Tx naïve | Male | 1795 | 17017 | 867 | 16804 | ND | ND | ND | ND | >90 | 96 | <20 |
| **CLLV66** | On Tx | Female | <100 | <100 | <100 | <100 | ND | ND | ND | ND | >90 | <20 | <20 |
| **CLLV67** | Off Tx CR | Male | 8617 | 105371 | 2917 | 40868 | ND | ND | ND | ND | 15.4 | 247 | 322 |
| **CLLV68** | Tx naïve | Male | 1000 | 14080 | 118 | 1516 | <100 | 1254 | 1990 | 12730 | >90 | <20 | <20 |

| | | | | | | | | | | | | |
|---|---|---|---|---|---|---|---|---|---|---|---|---|
| **CLLV69** | Tx naïve | Male | 36581 | >312500 | 7678 | 107963 | ND | ND | ND | ND | 4.2 | 611 | 772 |
| **CLLV70** | On Tx | Female | 1676 | 22342 | <100 | 386 | 146 | 3190 | 2059 | 14683 | >90 | <20 | <20 |
| **CLLV71** | On Tx | Male | <100 | <100 | <100 | <100 | ND | ND | ND | ND | >90 | <20 | <20 |
| **CLLV72** | Off Tx CR | Male | 4584 | 115885 | 1999 | 18279 | ND | ND | ND | ND | 32.9 | 195 | 498 |
| **CLLV73** | On Tx | Female | <100 | <100 | <100 | <100 | ND | ND | ND | ND | >90 | <20 | <20 |
| **CLLV74** | On Tx | Male | 188 | 8745 | 230 | 767 | <100 | 2989 | 121 | 4230 | >90 | <20 | <20 |
| **CLLV75** | On Tx | Male | <100 | <100 | <100 | <100 | ND | ND | ND | ND | >90 | <20 | <20 |
| **CLLV76** | Tx naïve | Female | 2733 | 34931 | 744 | 15249 | ND | ND | ND | ND | 87.4 | 61 | <20 |
| **CLLV77** | On Tx | Female | 2128 | 26777 | 454 | 16099 | ND | ND | ND | ND | >90 | 65 | 80 |
| **CLLV78** | Tx naïve | Male | 2903 | 44698 | 281 | 451 | 427 | 798 | 2254 | 25803 | >90 | <20 | <20 |
| **CLLV79** | Tx naïve | Female | 3929 | 62799 | 212 | 654 | 210 | 6027 | 2898 | 43683 | >90 | <20 | <20 |
| **CLLV80** | Off Tx and R/R | Female | 62608 | >312500 | 16113 | >312500 | ND | ND | ND | ND | 7.3 | 2255 | 2604 |
| **CLLV81** | On Tx | Male | <100 | <100 | <100 | <100 | ND | ND | ND | ND | >90 | <20 | <20 |
| **CLLV82** | Tx naïve | Female | 1509 | 18125 | 209 | 1313 | ND | ND | ND | ND | >90 | 41 | <20 |
| **CLLV83** | On Tx | Male | <100 | <100 | <100 | <100 | ND | ND | ND | ND | >90 | <20 | <20 |
| **CLLV84** | Tx naïve | Female | 22404 | >312500 | 5557 | 76049 | ND | ND | ND | ND | 33.7 | 548 | 374 |
| **CLLV85** | Tx naïve | Female | 1384 | 17623 | 263 | 6252 | ND | ND | ND | ND | >90 | <20 | <20 |
| **CLLV86** | Tx naïve | Female | 1791 | 10231 | <100 | <100 | 396 | 9514 | 3186 | 54983 | >90 | <20 | <20 |
| **CLLV87** | Tx naïve | Male | <100 | <100 | <100 | <100 | ND | ND | ND | ND | >90 | <20 | <20 |
| **CLLV88** | Tx naïve | Female | 555 | 11537 | 169 | 1723 | ND | ND | ND | ND | >90 | <20 | <20 |
| **CLLV89** | Off Tx CR | Male | 1861 | 16261 | 284 | 5827 | ND | ND | ND | ND | >90 | <20 | <20 |
| **CLLV90** | Tx naïve | Male | 10348 | 92902 | 2163 | 15895 | ND | ND | ND | ND | 30.8 | 101 | 182 |
| **CLLV91** | Tx naïve | Female | 1371 | 16682 | <100 | <100 | ND | ND | ND | ND | >90 | 30 | 21 |
| **CLLV92** | On Tx | Female | <100 | <100 | <100 | <100 | ND | ND | ND | ND | >90 | <20 | <20 |
| **CLLV93** | Tx naïve | Female | 3695 | 70113 | 1864 | 18575 | ND | ND | ND | ND | 51.7 | 139 | 161 |
| **CLLV94** | Tx naïve | Male | 5006 | 94551 | 1176 | 15881 | ND | ND | ND | ND | >90 | 134 | 290 |
| **CLLV95** | Tx naïve | Female | 564 | 14560 | 219 | 2901 | ND | ND | ND | ND | >90 | 27 | 29 |

Assay sensitivity cut-off values for Spike and RBD were >100; for the D614G and Delta neutralization assays >20; and >90% for RBD/ACE2 binding.

SARS-CoV-2, Severe Acute Respiratory Syndrome Coronavirus-2; CLL, chronic lymphocytic leukemia; $EC_{50}$, half-maximal effective concentration; RBD, receptor binding domain; S1, spike subdomain 1; S2, spike subdomain 2; ACE2, angiotensin-converting enzyme-2; $ID_{50}$, half-maximal neutralizing titers; HC, healthy control; ND, no data; Tx, treatment; CR, clinical remission, R/R, relapsed refractory; NA, not applicable.
