# Supplementary material for: Exposure of progressive immune dysfunction by SARS-CoV-2 mRNA vaccination in patients with chronic lymphocytic leukemia: A prospective cohort study
Source: PLoS Med. 2023 Jun 29;20(6):e1004157. doi: 10.1371/journal.pmed.1004157 (PMC10309642; doi:10.1371/journal.pmed.1004157)
Supplement: S3 Table — (PDF) [file pmed.1004157.s008.pdf]

S3 Table. Humoral immune responses for SARS-CoV-2 vaccinated healthy controls and CLL patients by disease/treatment status.

		HC	Total CLL	Total CLL responders	Tx naïve	On Tx	Off Tx CR	Off Tx and R/R
**Spike**	Number	30	95	65	45	34	9	7
EP	Responders	30	65	NA	43	9	9	4
	Response rate (%)	100	68	NA	96	27	100	57
EC_{50}	Median	15355	662	2128	2733	<100	2740	305
	95% CI of median							
	Lower confidence limit	9754	188	1384	1000	<100	870	100
	Upper confidence limit	23979	1791	3175	3800	<100	19885	62608
**RBD**	Number	30	95	51	45	34	9	7
EP	Responders	30	51	NA	35	4	9	3
	Response rate (%)	100	54	NA	78	12	100	43
EC_{50}	Median	3530	118	549	263	<100	708	<100
	95% CI of median							
	Lower confidence limit	2396	100	253	169	<100	247	<100
	Upper confidence limit	6343	212	1185	1172	<100	3338	16113
**D614G**	Number	30	95	40	45	34	9	7
Neut ID_{50}	Responders	29	40	NA	28	1	8	3
	Response rate (%)	97	42	NA	62	3	89	43
	Median	464	<20	174	30	<20	65	<20
	95% CI of median							
	Lower confidence limit	368	<20	69	<20	<20	21.8	<20
	Upper confidence limit	1046	22	308	140	<20	508	2255
**Delta**	Number	30	93	35	45	32	9	7
Neut ID_{50}	Responders	28	35	NA	24	1	7	3
	Response rate (%)	93	38	NA	53	3	78	43
	Median	346	<20	276	20	<20	156	<20
	95% CI of median							
	Lower confidence limit	171	<20	123	<20	<20	<20	<20
	Upper confidence limit	651	<20	374	123	<20	498	2604
**ACE2/RBD**	Number	30	95	28	45	34	9	7
**binding**	Responders	30	28	NA	19	0	7	2
**(%)**	Response rate (%)	100	30	NA	42	0	78	29
	Median	16	>90	37	>90	>90	59	>90
	95% CI of median							
	Lower confidence limit	6	>90	15	62	>90	15	7
	Upper confidence limit	22	>90	53	>90	>90	>90	>90

Assay sensitivity cut-off values for Spike and RBD were >100; for the D614G and Delta neutralization assays >20; and >90% for RBD/ACE2 binding. For spike and RBD, responders were determined by the detection of EP titer reactivity.

SARS-CoV-2, severe acute respiratory syndrome coronavirus-2; CLL, chronic lymphocytic leukemia; HC, healthy control; Tx, treatment; CR, clinical remission, R/R, relapsed refractory; EP, endpoint; NA, not applicable; EC_{50}, half-maximal effective concentration; CI, confidence interval; RBD, receptor binding domain; Neut ID_{50}, half-maximal neutralizing titers; ACE2, angiotensin-converting enzyme-2.
