# Supplementary material for: Exposure of progressive immune dysfunction by SARS-CoV-2 mRNA vaccination in patients with chronic lymphocytic leukemia: A prospective cohort study
Source: PLoS Med. 2023 Jun 29;20(6):e1004157. doi: 10.1371/journal.pmed.1004157 (PMC10309642; doi:10.1371/journal.pmed.1004157)
Supplement: S4 Table — (PDF) [file pmed.1004157.s009.pdf]

S4 Table. Associations of serologic responses for SARS-CoV-2 vaccinated healthy controls and CLL patients by disease/ treatment status.

	Response Rate				Titer			
		**HC vs. Total CLL**	**HC vs. CLL Tx naïve**	**HC vs. CLL Off Tx CR**		**HC vs. Total CLL**	**HC vs. CLL Tx naïve**	**HC vs. CLL Off Tx CR**
**Spike**[1]	Fisher's *p*-value<br>OR<br>95% CI	<0.001<br>+infinity<br>3.7, +infinity	0.5<br>+infinity<br>0.3, +infinity	>0.99	Mann-Whitney *p*-value	<0.001	<0.001	0.01
**RBD**[1]	Fisher's *p*-value<br>OR<br>95% CI	<0.001<br>+infinity<br>7.0, +infinity	0.005<br>+infinity<br>2.2, +infinity	>0.99	Mann-Whitney *p*-value	<0.001	<0.001	0.02
**D614G** Neut ID_{50}	Fisher's *p*-value<br>OR<br>95% CI	<0.001<br>40<br>6.7, 416	<0.001<br>18<br>2.9, 190	0.4<br>3.6<br>0.2, 71	Mann-Whitney *p*-value	<0.001	<0.001	0.02
**Delta** Neut ID_{50}	Fisher's *p*-value<br>OR<br>95% CI	<0.001<br>48<br>8.0, 501	<0.001<br>25<br>3.7, 272	0.2<br>4.0<br>0.5, 28	Mann-Whitney *p*-value	<0.001	<0.001	0.2
**ACE2/RBD binding (%)**	Fisher's *p*-value<br>OR<br>95% CI	<0.001<br>+infinity<br>19, +infinity	<0.001<br>+infinity<br>9.6, +infinity	0.05<br>+infinity<br>1.7, +infinity	Mann-Whitney *p*-value	<0.001	<0.001	0.01

[1]For Spike and RBD comparisons, response rates were calculated based on endpoint titers. Antibody titers were compared using median EC_{50} values.

Binary outcomes were calculated by Fisher's exact test. Assay sensitivity cut-off values for Spike and RBD were >100; for the D614G and Delta neutralization assays >20; and >90% for RBD/ACE2 binding. *p*-values for differences in medians were calculated by Dunn's multiple comparisons test.

SARS-CoV-2, severe acute respiratory syndrome coronavirus-2; CLL, chronic lymphocytic leukemia; HC, healthy control; Tx, treatment; CR, clinical remission; EC_{50}, half-maximal effective concentration; OR, odds ratio; CI, confidence interval; RBD, receptor binding domain; Neut ID_{50}, half-maximal neutralizing titers; ACE2, angiotensin-converting enzyme-2.
