# Supplementary material for: Exposure of progressive immune dysfunction by SARS-CoV-2 mRNA vaccination in patients with chronic lymphocytic leukemia: A prospective cohort study
Source: PLoS Med. 2023 Jun 29;20(6):e1004157. doi: 10.1371/journal.pmed.1004157 (PMC10309642; doi:10.1371/journal.pmed.1004157)
Supplement: S5 Table — (PDF) [file pmed.1004157.s010.pdf]

S5 Table. Univariate analysis of serologic responses for SARS-CoV-2 vaccinated CLL patients by disease/treatment status.

		**Naïve vs. On Tx**	**Naïve vs. Off Tx CR**	**Naïve vs. Off Tx and R/R**	**On Tx vs. Off Tx CR**	**On Tx vs. Off Tx and R/R**	**Off Tx CR vs. Off Tx and R/R**
**Spike**	Fisher's *p*-value	<0.001	>0.99	0.01	<0.001	0.2	0.06
EP	OR	60	<0.001	16	<0.001	0.3	+infinity
	95% CI	12, 269	<0.001, 11	2.4, 101	<0.001, 0.2	0.06, 1.2	1.3, +infinity
EC_{50}	Dunn's *p*-value	<0.001	>0.99	>0.99	<0.001	0.4	0.6
**RBD**	Fisher's *p*-value	<0.001	0.2	0.08	<0.001	0.08	0.02
EP	OR	26	<0.001	4.7	<0.001	0.2	+infinity
	95% CI	7.1, 78	<0.001, 1.8	1.1, 20	<0.001, 0.09	0.03, 1	1.4, +infinity
EC_{50}	Dunn's *p*-value	<0.001	0.7	0.6	<0.001	>0.99	0.08
**D614G**	Fisher's *p*-value	<0.001	0.2	0.4	<0.001	0.01	0.1
Neut ID_{50}	OR	54	0.2	2.2	0.004	0.04	11
	95% CI	7.8, 573	0.02, 1.3	0.5, 9.4	<0.001, 0.08	0.003, 0.4	0.8, 143
	Dunn's *p*-value	<0.001	>0.99	>0.99	<0.001	0.2	>0.99
**Delta**	Fisher's *p*-value	<0.001	0.3	0.7	<0.001	0.01	0.3
Neut ID_{50}	OR	35	0.3	1.5	0.009	0.04	4.7
	95% CI	5.2, 376	0.06, 1.5	0.4, 6.5	<0.001, 0.1	0.003, 0.4	0.5, 32
	Dunn's *p*-value	<0.001	0.5	>0.99	<0.001	0.2	>0.99
**ACE2/RBD**	Fisher's *p*-value	<0.001	0.07	0.7	<0.001	0.03	0.1
**binding**	OR	+infinity	0.2	1.8	<0.001	<0.001	8.8
**(%)**	95% CI	5.9, +infinity	0.04, 1	0.3, 10	<0.001, 0.06	<0.001, 0.4	0.9, 62
	Dunn's *p*-value	<0.001	0.3	>0.99	<0.001	>0.99	0.1

Binary outcomes were calculated by Fisher's exact test. Assay sensitivity cut-off values for Spike and RBD were >100; for the D614G and Delta neutralization assays >20; and >90% for RBD/ACE2 binding. For spike and RBD, responders were determined by the detection of EP titer reactivity. *p*-values for differences in medians were calculated by Dunn's multiple comparisons test.

SARS-CoV-2, severe acute respiratory syndrome coronavirus-2; CLL, chronic lymphocytic leukemia; Tx, treatment; CR, clinical remission; R/R, relapsed refractory; EP, endpoint; EC_{50}, half-maximal effective concentration; OR, odds ratio; CI, confidence interval; RBD, receptor binding domain; Neut ID_{50}, half-maximal neutralizing titers; ACE2, angiotensin-converting enzyme-2.
