# Supplementary material for: Exposure of progressive immune dysfunction by SARS-CoV-2 mRNA vaccination in patients with chronic lymphocytic leukemia: A prospective cohort study
Source: PLoS Med. 2023 Jun 29;20(6):e1004157. doi: 10.1371/journal.pmed.1004157 (PMC10309642; doi:10.1371/journal.pmed.1004157)
Supplement: S6 Table — (PDF) [file pmed.1004157.s011.pdf]

**S6 Table. Clinical and disease characteristics of participants included in studies of T cell immunity.**

| **Characteristic** | **Healthy controls (n=21)** | **CLL (n=36)** |
|---|---|---|
| Age in years, median (IQR) | 57 (41-69) | 72 (63-78) |
| Age ≥65 years, number (%) | 7 (33) | 27 (75) |
| Sex, male, number (%) | 14 (67) | 18 (50) |
| **mRNA vaccine** | | |
| Pfizer-BioNTech BNT162b2 | 18 (86) | 27 (75) |
| Moderna mRNA-1273 | 3 (14) | 9 (25) |
| Days from second vaccination to testing, median (IQR) | 36 (30-57) | 37 (27-69) |
| **Rai Stage[1], number (%)** | | |
| 0-II | | 20/26 (77) |
| III-IV | | 6/26 (23) |
| **Disease/treatment status, number (%)** | | |
| Treatment-naive | | 20 (55) |
| Active-therapy | | 8 (22) |
| Off-therapy in remission | | 2 (6) |
| Off-therapy in relapse | | 6 (17) |
| **Molecular and phenotypic biomarkers, number (%)** | | |
| *IGHV*, mutated | | 25/32 (78) |
| CD38 (≥20%) | | 9/34 (27) |
| **FISH, number (%)** | | |
| Normal | | 6/36 (17) |
| del(13q) | | 21/36 (57) |
| Trisomy 12 | | 5/36 (14) |
| del(11q) | | 2/36 (6) |
| del(17p) | | 2/36 (6) |
| **IVIg therapy** | | |
| Number (%) | | 8/36 (22) |
| **Laboratory parameters, median (IQR)** | | |
| Absolute lymphocyte count ($10^9$/L) | | 26 (6.5-61) |
| $\beta$2-microglobulin (mg/L) | | 2.2 (1.9-3.2) |
| IgM (mg/dL) | | 35 (23-68) |
| IgG (mg/dL) | | 713 (530-916) |
| IgA (mg/dL) | | 102 (64-160) |

[1]Determined for treatment-naïve and patients off therapy in relapse.

CLL, chronic lymphocytic leukemia; IQR, interquartile range; *IGHV*, immunoglobulin heavy chain variable gene; CD38, cluster of differentiation 38; FISH, fluorescence *in situ* hybridization; del, deletion; IVIg, intravenous immunoglobulin; IgM, immunoglobulin M; IgG, immunoglobulin G; IgA, immunoglobulin A.
