# Supplementary material for: Exposure of progressive immune dysfunction by SARS-CoV-2 mRNA vaccination in patients with chronic lymphocytic leukemia: A prospective cohort study
Source: PLoS Med. 2023 Jun 29;20(6):e1004157. doi: 10.1371/journal.pmed.1004157 (PMC10309642; doi:10.1371/journal.pmed.1004157)
Supplement: S7 Table — (PDF) [file pmed.1004157.s012.pdf]

S7 Table. Subset frequencies, SARS-CoV-2 S peptide-specific AIM responses, and effector functions in healthy control and CLL participant T cells.

A.

ID	Status	Gender	Total T cells	CD4 T cell frequencies					CD8 T cell frequencies				
				Total	Naïve and memory subsets				Total	Naïve and memory subsets			
			CD19- CD3+	CD3+ CD4+	(Tcm) Q1: CD45RA- CCR7+	(Naive) Q2: CD45R+ CCR7+	(Terma) Q3: CD45RA+ CCR7-	(Tem) Q4: CD45RA- CCR7-	CD3+ CD8+	(Tcm) Q1: CD45RA- CCR7+	(Naive) Q2: CD45RA+ CCR7+	(Terma) Q3: CD45RA+ CCR7-	(Tem) Q4: CD45RA- CCR7-
**HC2**	Healthy	Female	80.7	80.2	30.9	49.8	2.4	16.9	17.1	10	27.1	38	24.8
**HC5**	Healthy	Male	74.1	79.1	24.9	36.5	6.16	32.5	10.3	8	30.1	21.5	40.4
**HC8**	Healthy	Male	77.9	77.2	25.7	37.2	6.69	30.4	16.3	2.95	1.85	74.1	21.1
**HC9**	Healthy	Female	82.1	91.3	36.6	46.6	2.96	13.8	4.92	8.47	17.8	48.5	25.2
**HC11**	Healthy	Male	85.6	87.7	23.1	60.9	2.33	13.6	7.69	9.01	23.4	21.5	46
**HC13**	Healthy	Male	40.9	92.9	30.4	36.5	4.04	29.1	3.32	3.22	14.8	56.6	25.4
**HC14**	Healthy	Female	80.3	80.2	17.7	44.4	4.56	33.4	13.8	5.09	20.2	32.9	41.8
**HC15**	Healthy	Female	86.9	92.9	27.1	53.1	2.6	17.2	3.31	18.5	47.7	6.68	27.1
**HC16**	Healthy	Female	90.1	81.5	28.2	33.9	5.04	32.9	13.4	18.5	39.3	12.2	30
**HC17**	Healthy	Female	90	76.2	26.7	36.8	6.08	30.4	18.3	7.33	22.1	38.3	32.2
**HC18**	Healthy	Male	80.2	86	22.3	23.2	10.7	43.8	7.17	9.86	26.1	18.9	45.1
**HC20**	Healthy	Male	76.7	82.1	27.7	38.8	5.5	28	8.4	6.26	45.2	21.7	26.9
**HC21**	Healthy	Male	76.5	77.4	26.8	41.6	8.2	23.4	7.27	13.8	31.9	9.45	44.9
**HC22**	Healthy	Male	80.3	65.1	23.5	46.5	6.67	23.3	19.7	32.6	28.7	14.6	24.1
**HC23**	Healthy	Female	89.4	72.3	25.4	39.8	7.94	26.8	21.2	9.66	27.2	17.9	45.3
**HC24**	Healthy	Male	78.7	66.8	30.4	50.6	2.36	16.6	22.8	7.69	53	4.56	34.7
**HC25**	Healthy	Male	80.4	53.1	22.3	24.7	5.42	47.5	16.7	7.19	12.8	9.25	70.8
**HC26**	Healthy	Male	60.9	82.5	19.7	42.4	9.66	28.2	4.49	6.69	45.9	5.42	42
**HC28**	Healthy	Male	79.1	72.9	23.2	42.9	3.94	30	18.3	10.4	3.77	51.6	34.3
**HC30**	Healthy	Male	76	86.3	29.6	46.4	3.8	20.2	5.62	17.7	19.7	3.63	59
**HC31**	Healthy	Male	84.6	40.6	17.2	26.7	11.2	44.9	8.65	9.95	18.4	32.6	39
**CLLV1**	On Tx	Male	81.1	81.4	10.4	50.5	15.4	23.7	11.8	1.99	8.55	72.5	17
**CLLV2**	Tx naïve	Male	7.79	82.1	45.4	26.2	1.5	27	11.4	13.9	21	35.9	29.1
**CLLV6**	On Tx	Female	46.6	75.3	37.5	16	15.9	30.6	9.12	11.1	10.7	37.5	40.7
**CLLV12**	Tx naïve	Male	13.2	67.7	37.7	18.3	4.32	39.7	28.1	2.88	1.57	39.2	56.4
**CLLV21**	Tx naïve	Male	33.6	68.5	19.1	25.5	15.1	40.4	16.3	2.52	2.72	59.4	35.3
**CLLV23**	Tx naïve	Female	9.91	85.6	40.4	32.9	2.48	24.3	9.12	16.1	11.4	26.9	45.6
**CLLV27**	Tx naïve	Female	15.1	68.1	16.2	50.9	7.62	25.3	23.2	2.03	11.7	66.5	19.7

**CLLV30**	Tx naïve	Male	2.12	88	19.6	44.3	7.08	29	8.37	1.26	10.6	32.6	55.6
**CLLV31**	Off Tx and R/R	Female	7.78	45.2	19.7	3.3	4.09	72.9	44.6	2.9	1.45	52.2	43.4
**CLLV33**	Tx naïve	Male	37.9	42.4	38.7	31.7	6.67	23	37.8	1.91	0.6	71	26.5
**CLLV34**	Tx naïve	Female	19.5	76.1	30.7	5.89	8.08	55.3	11.1	13.5	1.81	26.7	57.9
**CLLV35**	Off Tx CR	Female	42.7	72	19.3	48.5	5.05	27.2	20.3	5.16	45	12.3	37.5
**CLLV36**	Tx naïve	Female	3.3	63.5	51.6	21.5	4.05	22.8	18.3	27.3	10.7	5.81	56.2
**CLLV40**	Off Tx and R/R	Female	0.98	51.8	20.8	14.5	11	53.7	39.8	6.29	1	22	70.7
**CLLV47**	Off Tx CR	Female	55.3	74.3	21.3	9.52	9.27	59.9	21.2	18	6.8	16	59.2
**CLLV48**	Tx naïve	Female	16	75.5	14.9	54.2	7.98	23	21.5	2.04	2.33	10	85.6
**CLLV49**	Off Tx and R/R	Male	2.45	64.5	41.9	3.33	3.88	50.9	25.6	19	1.08	25.4	54.6
**CLLV50**	On Tx	Female	6.36	54.5	17.2	15.5	6.11	61.2	37.8	3.8	3.46	28.8	63.9
**CLLV51**	On Tx	Male	80.1	88.1	12.9	33.1	17.9	36.2	5.85	5.04	25.3	27.1	42.5
**CLLV54**	Off Tx and R/R	Female	4.04	76.1	19.1	70.1	2.51	8.33	6.2	8.61	26	26.5	39
**CLLV55**	Tx naïve	Male	3.78	34.3	30.8	14.5	8.49	46.2	42	11.7	7.08	8.23	72.9
**CLLV57**	Tx naïve	Female	1.64	54	40.1	41.9	2.31	15.6	32.6	19.5	8.22	18.6	53.7
**CLLV58**	Tx naïve	Female	8.99	75	22.7	46.9	10.5	19.8	15.1	8.42	53.2	30.6	7.81
**CLLV59**	Off Tx and R/R	Male	2.34	88.1	29.7	39.3	3.78	27.1	7.87	11.8	24.4	12.2	51.6
**CLLV60**	Tx naïve	Male	20.9	51.4	29	16.1	6.57	48.4	29.1	5.1	3.18	27	64.7
**CLLV61**	On Tx	Male	49	94.5	20.6	27.7	13.5	38.2	1.47	14.6	8.21	14.9	62.3
**CLLV62**	Tx naïve	Male	12.4	79.6	21	33.3	9.63	36	12.5	11.6	5.41	36.6	46.3
**CLLV64**	On Tx	Male	72.2	47	26.8	33	4.8	35.4	32.1	5.4	6.73	12.8	75
**CLLV65**	Tx naïve	Male	9.94	88.2	21.6	57.3	2.85	18.3	9.1	4.85	7.67	49.5	37.9
**CLLV69**	Tx naïve	Male	33.4	77.6	25.2	53.9	3.53	17.4	14.9	12.4	14.5	19.8	53.3
**CLLV70**	On Tx	Female	1.48	71	10.8	80.3	2.4	6.45	24.7	1.92	23.2	41.7	33.2
**CLLV75**	On Tx	Male	5.32	49.9	14.6	13.6	10	61.7	42.1	6.54	1.68	14	77.8
**CLLV78**	Tx naïve	Male	11.3	59.2	24.4	27.2	5.13	43.3	14.8	2.36	7.13	38.1	52.4
**CLLV80**	Off Tx and R/R	Female	4.44	56.4	33.4	15.9	3.59	47.1	39.9	5.84	1.07	20.6	72.4
**CLLV84**	Tx naïve	Female	14.4	61.9	36.8	16.9	5.55	40.8	31.7	8.26	3.79	26.9	61.1
**CLLV86**	Tx naïve	Female	34.7	66	32.2	41.8	2.05	24	27.7	15.1	14	36.2	34.6

Values shown for T cell subpopulations indicate frequencies (%) as determined by flow cytometry analysis.

SARS-CoV-2, Severe Acute Respiratory Syndrome Coronavirus-2; S, spike; AIM, activation induced marker; CLL, chronic lymphocytic leukemia; HC, healthy control; CD19, cluster of differentiation 19; CD3, cluster of differentiation 3; CD4, cluster of differentiation 4; N, naïve; CM, central memory; EM, effector memory; CD45RA, cluster of differentiation 45 including the A protein region; EMRA, effector memory CD45RA; CCR7, C-C chemokine receptor type 7; CD8, cluster of differentiation 8; Tx, treatment; CR, clinical remission, R/R, relapsed refractory.

B.

ID	N-specific T cell subset AIM responses			
	AIM responders			
	CD4	cTfh	CD8	SUM (N)
**HC2**	0	0	0	0
**HC5**	0	0	0	0
**HC8**	0	0	0	0
**HC9**	0	0	0	0
**HC11**	0	0	0	0
**HC13**	0	0	0	0
**HC14**	0	0	0	0
**HC15**	0	0	0	0
**HC16**	0	0	0	0
**HC17**	0	0	0	0
**HC18**	0	0	0	0
**HC20**	0	0	0	0
**HC21**	0	0	0	0
**HC22**	0	0	0	0
**HC23**	0	0	1	1
**HC24**	0	0	0	0
**HC25**	0	0	0	0
**HC26[a]**	ND	ND	ND	ND
**HC28**	0	0	0	0
**HC30[a]**	ND	ND	ND	ND
**HC31[a]**	ND	ND	ND	ND
**CLLV1**	0	0	0	0
**CLLV2**	0	0	0	0
**CLLV6**	0	0	0	0
**CLLV12**	0	0	0	0
**CLLV21**	0	0	0	0
**CLLV23**	0	0	0	0
**CLLV27**	0	0	0	0
**CLLV30**	0	0	0	0
**CLLV31**	0	0	0	0
**CLLV33**	0	0	0	0
**CLLV34**	1	0	0	1
**CLLV35**	0	0	0	0
**CLLV36**	0	0	0	0

**CLLV40**	0	0	0	0
**CLLV47**	0	0	0	0
**CLLV48**	0	0	0	0
**CLLV49**	1	0	0	1
**CLLV50**	0	0	0	0
**CLLV51**	0	0	0	0
**CLLV54**	0	0	0	0
**CLLV55**	0	0	0	0
**CLLV57**	0	0	0	0
**CLLV58**	0	0	0	0
**CLLV59**	1	0	0	1
**CLLV60**	0	0	0	0
**CLLV61**	0	0	0	0
**CLLV62**	0	0	0	0
**CLLV64**	0	0	0	0
**CLLV65**	0	0	0	0
**CLLV69**	0	0	0	0
**CLLV70**	0	0	0	0
**CLLV75**	0	0	0	0
**CLLV78**	0	0	0	0
**CLLV80**	0	0	0	0
**CLLV84**	0	0	0	0
**CLLV86**	0	0	0	0

[a]Material was not available to run the assay on these samples.

Values of "1" indicate the presence and "0" the absence of a response.

N, nucleocapsid; AIM, activation-induced marker; CD4, cluster of differentiation 4; CTfh, circulating T follicular helper; CD8, cluster of differentiation 8; CLL, chronic lymphocytic leukemia; HC, healthy control; ND, no data.

C.

ID	S-specific T cell subset AIM responses							
	AIM responders					Response frequencies		
	CD4	cTfh	CD8	Positive (S)	SUM (S)	CD4	cTfh	CD8
**HC2**	1	1	1	1	3	0.35	2.13	0.28
**HC5**	1	0	0	1	1	0.06	0.00	0.09
**HC8**	1	1	1	1	3	0.83	2.81	0.20
**HC9**	1	0	0	1	1	0.09	0.61	0.00
**HC11**	1	1	0	1	2	0.31	2.74	0.00
**HC13**	0	0	0	0	0	0.00	0.59	0.00
**HC14**	1	1	0	1	2	0.28	2.13	0.15
**HC15**	1	1	1	1	3	0.80	4.35	0.07
**HC16**	1	1	1	1	3	1.44	1.31	0.44
**HC17**	1	1	1	1	3	0.46	0.57	0.20
**HC18**	1	0	1	1	2	0.49	0.18	0.90
**HC20**	1	1	0	1	2	0.24	0.13	0.03
**HC21**	1	1	1	1	3	0.22	0.34	0.51
**HC22**	1	1	1	1	3	0.26	0.43	0.17
**HC23**	1	1	1	1	3	1.83	3.01	0.19
**HC24**	1	1	0	1	2	0.34	0.54	0.11
**HC25**	1	1	0	1	2	0.81	0.98	0.09
**HC26**	1	1	0	1	2	0.52	1.38	0.00
**HC28**	0	1	0	1	1	0.10	1.64	0.30
**HC30**	0	1	1	1	2	0.28	0.78	0.14
**HC31**	0	0	0	0	0	0.14	0.41	0.01
**CLLV1**	1	1	1	1	3	0.135	2.040	0.401
**CLLV2**	0	0	0	0	0	0.000	0.000	0.000
**CLLV6**	0	0	0	0	0	0.010	0.000	0.000
**CLLV12**	1	0	0	1	1	0.360	0.038	0.023
**CLLV21**	0	0	0	0	0	0.000	0.000	0.000
**CLLV23**	0	0	0	0	0	0.030	0.000	0.160
**CLLV27**	0	0	0	0	0	0.015	0.000	0.008
**CLLV30**	0	0	0	0	0	0.000	0.000	0.000
**CLLV31**	0	0	0	0	0	0.000	0.000	0.000
**CLLV33**	0	0	0	0	0	0.021	0.000	0.051
**CLLV34**	0	0	0	0	0	0.000	0.000	0.090
**CLLV35**	0	0	0	0	0	0.031	0.000	0.059
**CLLV36**	0	0	0	0	0	0.000	0.000	0.500

**CLLV40**	0	0	0	0	0	0.000	0.000	0.110
**CLLV47**	0	0	0	0	0	0.000	0.140	0.038
**CLLV48**	0	0	0	0	0	0.061	0.680	0.000
**CLLV49**	1	0	0	1	1	0.150	1.230	0.180
**CLLV50**	0	0	0	0	0	0.000	0.000	0.000
**CLLV51**	0	0	0	0	0	0.038	0.920	0.000
**CLLV54**	0	0	0	0	0	0.065	0.000	0.000
**CLLV55**	1	1	0	1	2	0.554	2.600	0.079
**CLLV57**	0	0	0	0	0	0.035	0.240	0.000
**CLLV58**	1	1	1	1	3	0.349	2.170	0.352
**CLLV59**	1	0	0	1	1	0.092	0.930	0.053
**CLLV60**	0	0	0	0	0	0.000	0.000	0.050
**CLLV61**	0	0	0	0	0	0.039	0.110	0.000
**CLLV62**	0	0	0	0	0	0.430	0.000	0.042
**CLLV64**	1	0	0	1	1	0.140	0.000	0.033
**CLLV65**	0	0	0	0	0	0.006	0.060	0.000
**CLLV69**	1	1	0	1	2	0.720	2.240	0.060
**CLLV70**	0	0	0	0	0	0.040	2.820	0.000
**CLLV75**	0	0	1	1	1	0.069	0.130	0.085
**CLLV78**	1	1	0	1	2	0.456	1.960	0.106
**CLLV80**	0	0	0	0	0	0.030	0.000	0.009
**CLLV84**	1	1	0	1	2	0.139	0.390	0.000
**CLLV86**	0	0	1	1	1	0.170	0.820	0.173

Values of "1" indicate the presence and "0" the absence of a response.

Values shown for T cell subpopulation responses indicate frequencies (%) as determined by flow cytometry analysis.

S, spike; AIM, activation-induced marker; CD4, cluster of differentiation 4; CTfh, circulating T follicular helper; CD8, cluster of differentiation 8; CLL, chronic lymphocytic leukemia; HC, healthy control.

D.

ID	S-specific T cell subset effector functions of healthy control and CLL vaccinees															
	ICS T cell responders				CD4 T cells						CD8 T cells					
	CD4	CD8	Positive (S)	SUM (S)	IFNγ	TNFα	IL2	Grz-B	Perforin	PFS	IFNγ	TNFα	IL2	Grz-B	Perforin	PFS
**HC2**	1	1	1	2	0.021	0.064	0.070	0.000	0.002	0.241	0.135	0.016	0.073	0.122	0.170	0.197
**HC5**	1	0	1	1	0.031	0.065	0.069	0.000	0.000	0.233	0.080	0.020	0.035	0.019	0.000	0.157
**HC8**	1	1	1	2	0.007	0.017	0.119	0.001	0.003	0.241	0.000	0.000	0.165	0.000	0.000	0.121
**HC9**	1	0	1	1	0.010	0.063	0.021	0.002	0.002	0.176	0.090	0.000	0.000	0.020	0.000	0.161
**HC11**	1	1	1	2	0.019	0.031	0.032	0.000	0.003	0.239	0.230	0.119	0.000	0.000	0.036	0.182
**HC13**																
**HC14**[a]	ND	ND	ND	ND	ND	ND	ND	ND	ND	ND	ND	ND	ND	ND	ND	ND
**HC15**	1	1	1	2	0.011	0.050	0.101	0.000	0.000	0.272	0.140	0.036	0.123	0.005	0.013	0.234
**HC16**	1	1	1	2	0.015	0.086	0.129	0.000	0.000	0.250	0.156	0.097	0.123	0.004	0.013	0.293
**HC17**	1	1	1	2	0.059	0.162	0.090	0.000	0.000	0.237	0.202	0.085	0.000	0.010	0.024	0.264
**HC18**	1	1	1	2	0.102	0.210	0.190	0.000	0.006	0.274	0.182	0.072	0.000	0.029	0.020	0.244
**HC20**	1	1	1	2	0.263	0.032	0.295	0.000	0.000	0.342	0.295	0.000	0.381	0.003	0.008	0.242
**HC21**	1	1	1	2	0.147	0.164	0.136	0.000	0.001	0.308	0.273	0.019	0.000	0.053	0.113	0.262
**HC22**	1	1	1	2	0.065	0.069	0.054	0.000	0.002	0.329	0.148	0.030	0.061	0.000	0.000	0.187
**HC23**																
**HC24**	1	1	1	2	0.064	0.068	0.079	0.001	0.002	0.360	0.069	0.016	0.115	0.008	0.008	0.242
**HC25**	1	1	1	2	0.025	0.063	0.037	0.000	0.000	0.190	0.116	0.007	0.000	0.000	0.000	0.233
**HC26**[a]	ND	ND	ND	ND	ND	ND	ND	ND	ND	ND	ND	ND	ND	ND	ND	ND
**HC28**	1	1	1	2	0.000	0.006	0.138	0.000	0.000	0.172	0.090	0.030	0.174	0.046	0.050	0.177
**HC30**[a]	ND	ND	ND	ND	ND	ND	ND	ND	ND	ND	ND	ND	ND	ND	ND	ND
**HC31**																
**CLLV1**	1	1	1	2	0.000	0.021	0.462	0.000	0.000	0.216	0.124	0.237	0.097	0.006	0.009	0.303
**CLLV2**																
**CLLV6**																
**CLLV12**	0	0	0	0	0.000	0.040	0.041	0.000	0.000	0.132	0.015	0.002	0.034	0.028	0.013	0.071
**CLLV21**																
**CLLV23**																
**CLLV27**																
**CLLV30**																
**CLLV31**																
**CLLV33**																
**CLLV34**																
**CLLV35**																
**CLLV36**																
**CLLV40**																

**CLLV47**																
**CLLV48**																
**CLLV49**																
**CLLV50**																
**CLLV51**																
**CLLV54**																
**CLLV55**	0	1	1	1	0.130	1.300	0.400	0.000	0.031	0.176	0.570	0.440	0.000	0.000	0.046	0.198
**CLLV57**																
**CLLV58**	0	0	0	0	0.000	0.110	0.064	0.000	0.000	0.052	0.000	0.000	0.000	0.000	0.000	0.000
**CLLV59**																
**CLLV60**																
**CLLV61**																
**CLLV62**																
**CLLV64**	0	0	0	0	0.000	0.033	0.000	0.050	0.150	0.000	0.000	0.470	0.000	0.040	0.030	0.001
**CLLV65**																
**CLLV69[a]**	ND	ND	ND	ND	ND	ND	ND	ND	ND	ND	ND	ND	ND	ND	ND	ND
**CLLV70**																
**CLLV75[a]**	ND	ND	ND	ND	ND	ND	ND	ND	ND	ND	ND	ND	ND	ND	ND	ND
**CLLV78[a]**	ND	ND	ND	ND	ND	ND	ND	ND	ND	ND	ND	ND	ND	ND	ND	ND
**CLLV80**																
**CLLV84**	0	1	1	1	0.002	0.048	0.000	0.002	0.000	0.166	0.047	0.004	0.000	0.010	0.013	0.182
**CLLV86**	1	1	1	2	0.019	0.120	0.050	0.000	0.000	0.167	0.046	0.020	0.044	0.021	0.008	0.204

[a]Material was not available to run the assay on these samples.

Values of "1" indicate the presence and "0" the absence of a response. For values left blank, data are not applicable.

Values shown for T cell subpopulation responses indicate frequencies (%) as determined by flow cytometry analysis.

S, spike; CLL, chronic lymphocytic leukemia; ICS, intra-cellular staining; CD4, cluster of differentiation 4; CD8, cluster of differentiation 8; HC, healthy control; IFNγ, interferon gamma; TNFα, tumor necrosis factor alpha; IL2, interleukin 2; Grz-B, granzyme B; PFS, polyfunctionality score calculated by COMPASS; ND, no data.
